# PKP1 promotes lung cancer by modulating energy metabolism through stabilization of PFKP

**DOI:** 10.1186/s40364-025-00815-w

**Published:** 2025-09-01

**Authors:** Félix Ritoré-Salazar, Alberto M. Arenas, Ana M. Matia-González, Alessandra Zaza, Emil Aagaard Thomsen, Anne Bruun Rovsing, Jacob Giehm Mikkelsen, Nelida Ines Noguera, Pedro P. Medina

**Affiliations:** 1 Department of Biochemistry and Molecular Biology I. Faculty of Sciences, University of Granada, Granada, Spain; 2 GENYO, Centre for Genomics and Oncological Research, Pfizer/University of Granada/Andalusian Regional Government, PTS Granada, Granada, Spain; 3 Instituto de Investigación Biosanitaria (ibs.GRANADA), Granada, Spain; 4 Present address: Science for Life Laboratory, Division of Genome Biology, Department of Medical Biochemistry and Biophysics, Karolinska Institutet, Stockholm, Sweden; 5 Department of Medical and Surgical Sciences and Biotechnologies, University of Roma La Sapienza, Rome, Italy; 6 Santa Lucia Foundation, I.R.C.C.S, Via del Fosso di Fiorano, Rome, Italy; 7 Department of Biomedicine, Aarhus University, Aarhus, Denmark; 8 Department of Biomedicine and Prevention, University of Rome Tor Vergata, Rome, Italy

**Keywords:** Plakophilin-1, Phosphofructokinase, TRIM21, Glycolysis and OXPHOS

## Abstract

Lung cancer is the leading cause of cancer-related deaths worldwide, with lung squamous cell carcinoma (LUSC) lacking effective targeted therapies. Recent studies have identified Plakophilin-1 (PKP1) as one of the most differentially overexpressed genes in LUSC. This is particularly intriguing given that PKP1 is primarily known as a desmosomal component involved in cell adhesion, typically regarded as a tumor suppressor. To elucidate its biological role, we performed a genome-wide CRISPR knockout screening in PKP1-deficient models, revealing a strong dependence on mitochondrial metabolism. Metabolic assays further demonstrated that PKP1 loss significantly disrupts both mitochondrial function and glycolytic activity. In contrast, cells expressing PKP1 display a metabolically hyperactive phenotype, characterized by elevated oxygen consumption rate (OCR) and extracellular acidification rate (ECAR). Building on these findings, we found that PKP1 depletion selectively reduces platelet-type phosphofructokinase (PFKP) levels, a key rate-limiting enzyme in glycolysis, by enhancing its ubiquitination and subsequent degradation. Functional rescue experiments confirmed that PFKP mediates the proliferative role of PKP1. These findings suggest that PKP1 overexpression in LUSC promotes a hyperactive metabolic state binding to TRIM21 and preventing PFKP degradation, facilitating tumor progression. These effects were consistently observed across multiple LUSC cell lines, underscoring the robustness of the mechanism. These findings highlight a potential therapeutic vulnerability in LUSC metabolic regulation.

**Graphical Abstract:**

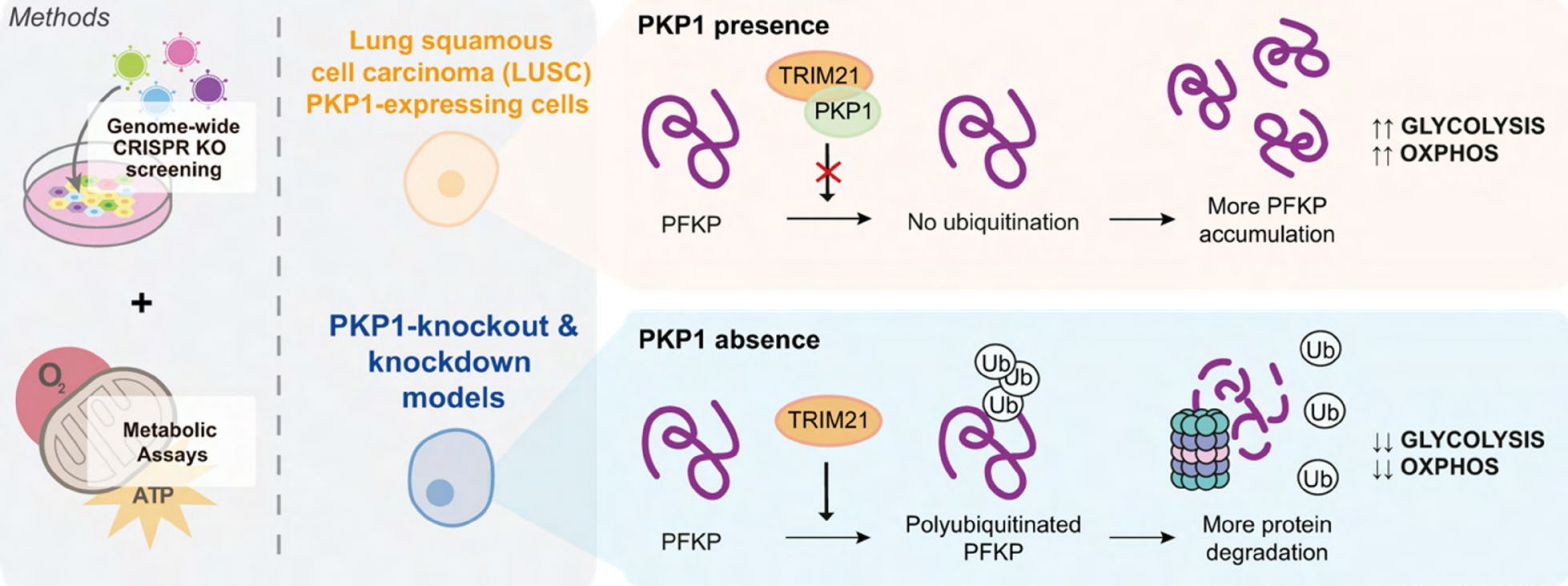

**Supplementary Information:**

The online version contains supplementary material available at 10.1186/s40364-025-00815-w.

**To the editor**.

## Introduction

Lung cancer remains the leading cause of cancer-related mortality worldwide [1], with non-small cell lung cancer (NSCLC) accounting for 85% of cases and small cell lung cancer (SCLC) representing the remaining 15%. NSCLC is primarily classified into two major subtypes: lung adenocarcinoma (LUAD) and lung squamous cell carcinoma (LUSC) [2], each characterized by distinct genetic profiles and treatment responses. Plakophilin-1 (PKP1) belongs to a group of proteins known as plakophilins, which are typically expressed in both stratified and simple epithelial cells, and plays a crucial role in maintaining desmosomal integrity. In a cancer context, desmosomes are traditionally associated with tumor-suppressive functions, such as promoting cell-cell adhesion and inhibiting cancer progression and metastasis [3]. However, this contrasts with observations in LUSC, where PKP1 is among the most overexpressed genes [4, 5]. Emerging evidence further suggests that PKP1 plays additional roles in cellular signaling [6] and gene expression regulation of key oncogenes, including c-Myc [7, 8]. Current lung cancer treatments, including surgery, chemotherapy, and radiotherapy, are standard but limited due to chemotherapy’s drug resistance and toxicity. Targeted therapies based on tumor mutational profiles show promise, yet significant genetic differences between LUSC and LUAD limit their application. Notably, mutations in common LUAD driver genes with available targeted therapies (e.g. *KRAS* and *EGFR*), are usually absent in LUSC [9]. Despite recent progress, effective targeted therapies for LUSC remain a challenge [10]. To address this gap, our study employed a genome-wide CRISPR knockout (KO) screen in LUSC cells with differential PKP1 expression. This approach aimed to elucidate PKP1’s role in lung cancer by identifying vulnerabilities linked to its loss, thereby uncovering novel functions in LUSC.

## Materials and methods

All detailed procedures are described in Supplemental text file 1.

## Results and discussion

**Fig. 1:**
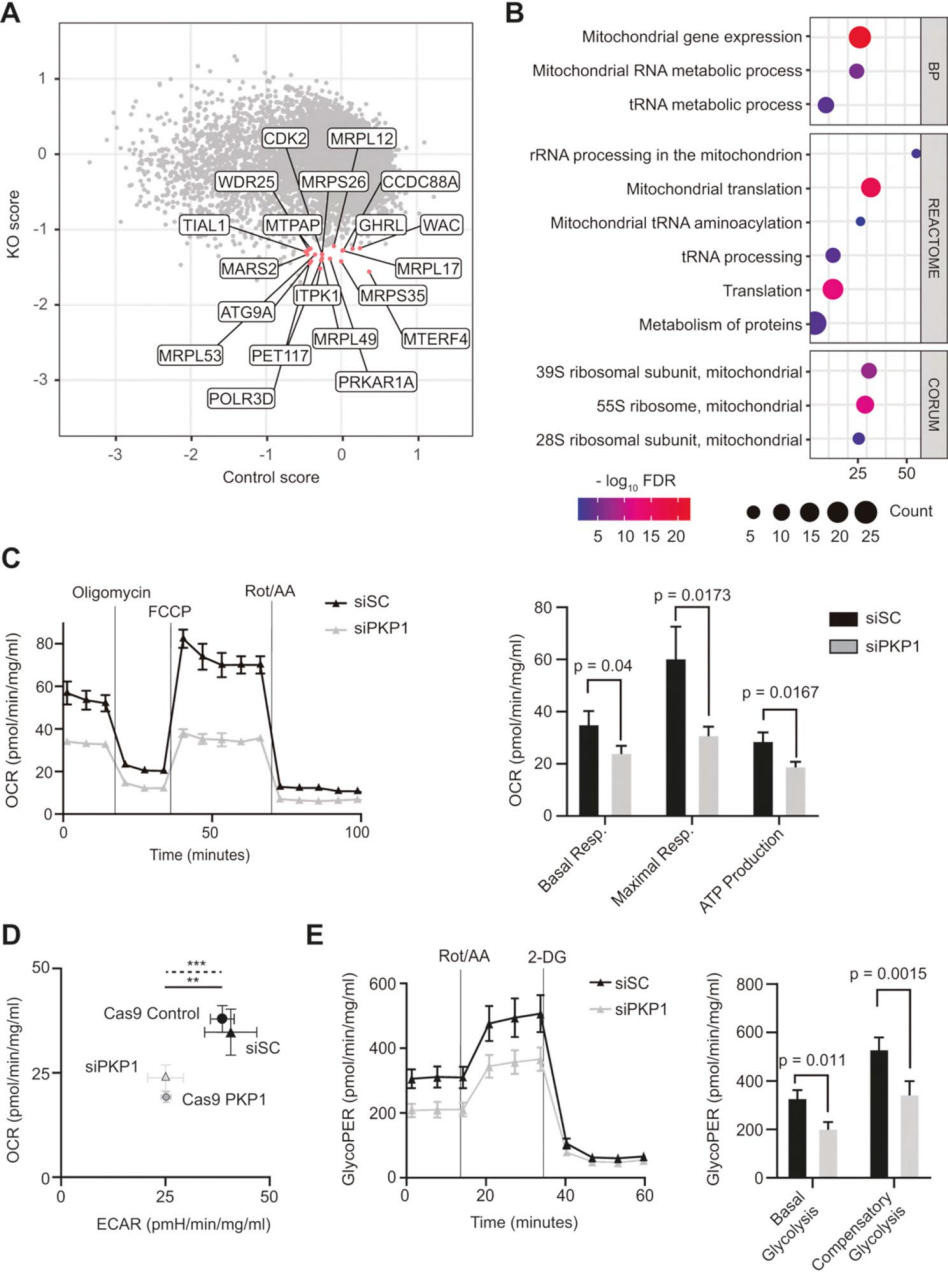
(**A**) Dot plot represents the gene essentiality score of all genes. Red labelled dots correspond to the top 20 genes within the lowest KO gene essentiality score (y-axis), and a neutral control gene essentiality score (x-axis) between − 0.5 and + 0.5. (**B**) Selection of GO (Biological Process - BP), REACTOME and CORUM terms overrepresented among the top 100 KO genes. The diameter of the circle is proportional to the number of genes, the colormap refers to the *-*log_10_ FDR. *X*-axis specifies the enrichment score. (**C**) **Left panel**: OCR profile at baseline and in response to oligomycin, carbonyl cyanide 4-(trifluoromethoxy) phenylhydrazone (FCCP), and antimycin A plus rotenone (Rot/AA) of SK-MES-1 KD model. **Right panel**: Bar plots show the quantification of basal respiration, maximal respiration and ATP production in the KD model. Results were normalized to total concentration of protein in each well. All experiments were conducted in triplicates and *P* values were calculated using an unpaired, two-tailed *t*-test. (**D**) Energy Map of basal OCR and basal ECAR in SK-MES-1 cells in KO and KD models, using the Seahorse Bioscience XF96 analyzer. Values represent mean ± SD (*n* = 3). MANOVA-test *P < 0.05; **P < 0.01; ***P < 0.001, solid line refers to the KO model comparison and dashed line to the silenced model. (**E**) GlycoPER profile at baseline and in response to Rot/AA and 2-deoxy-D-glucose (2-DG) of SK-MES-1 lacking PKP1 (left). Bar plots show quantification of basal and compensatory glycolysis in the KD model (right). Results were normalized to total concentration of protein of each well. All experiments were conducted in triplicates and *P* values were calculated using an unpaired, two-tailed *t*-test

First, we performed a genome-wide CRISPR KO screen in the parental SK-MES-1 cell line (LUSC cell line that endogenously expresses PKP1), and on two individual biallelic SK-MES-1 KO clones [8]. We then assigned a gene essentiality score to each gene by comparing sgRNA abundances between the baseline (plasmid distribution of sgRNA) and endpoint (day 21). We compared the gene essentiality score of the KO cells to the control cells and KO genes with the lowest essentiality score and ranging from − 0.5 to 0.5 in the control, were selected. The top hits of the CRISPR screening are composed mainly of mitochondrial ribosomal structural proteins (MRPs), such as MRPS35, MRPS26, MRPL49, or MRPL53 and other mitochondrial components like PET117 or MTERF4 (Fig. 1.A). Furthermore, functional enrichment analysis including Biological Process (BP), REACTOME gene sets and CORUM complexes were conducted using the top 100 KO scoring genes and consistently highlighted a significant enrichment of mitochondrial-related pathways (Fig. 1.B). These findings suggest that the lack of PKP1 affects mitochondrial function, potentially resulting in an impairment of the cellular metabolism. Dysregulated metabolism is included in the core of hallmarks of cancer [11], a concept pioneered by Otto Warburg [12], where tumors rely on glycolysis for energy production even in the presence of oxygen. This contrasts with normal cells, which preferentially rely on oxidative phosphorylation (OXPHOS) over glycolysis when oxygen is present. However, not every cancer cell undergoes the Warburg effect, since other metabolic profiles with active and fully functional mitochondria have been observed [13].

Next, we studied mitochondrial function (basal and maximal respiration, and ATP production) in PKP1-depleted LUSC cells in both KO and small interfering RNA (siRNA) knockdown (KD) models. Intriguingly, in both experimental models, we found that the oxygen consumption rate (OCR) was reduced to their respective PKP1-expression control cells in basal and maximal respiration as well as ATP production, all indicators of mitochondrial functions (Fig. 1.**C** and S.1 A). These results further highlight that PKP1 plays a significant role in maintaining efficient energy production and mitochondrial function. Although its silencing does not appear to impact mitochondrial abundance, as evidenced by the unaltered expression of the mitochondrial marker TOM20 (Fig S.1B), PKP1 loss still results in reduced mitochondrial function.

To gain further insight on cancer cells metabolic status upon PKP1 depletion we analyzed the glycolysis activity by measuring the extracellular acidification rate (ECAR). Energy map analysis (Fig. 1.D) revealed that PKP1 loss compromises both mitochondrial respiration and glycolytic activity in both KD and KO models. PKP1-expressing cells exhibited a highly metabolically active phenotype, characterized by elevated OCR and ECAR values, which reflect enhanced metabolic plasticity through the simultaneous activation of both OXPHOS and glycolysis [14]. In contrast, PKP1-deficient cells displayed significantly reduced OCR and ECAR, indicative of a globally impaired metabolic state. Furthermore, we observed a significant reduction in the glycolytic Proton Efflux Rate (glycoPER) in PKP1-deficient cells during both basal and compensatory glycolysis phases in the KD (Fig. 1.**E**) and KO (Fig S.2 A) models. Our findings highlight the metabolic heterogeneity of cancer [13], as PKP1-expressing SK-MES-1 cells deviate from the typical Warburg effect exhibiting high rates of both glycolysis and OXPHOS. This was further confirmed as both glycolytic ATP (glycoATP) and mitochondrial ATP (mitoATP) production are impaired (Fig S.2B).

In parallel, we conducted a reanalysis of a publicly available microarray dataset (GSE106770) in which PKP1 was silenced in SK-MES-1 cells [8]. Gene set enrichment analysis revealed a strong enrichment of metabolic pathways, with glycolysis emerging as one of the most significantly altered categories (Fig S.3). This independent transcriptomic evidence provided an initial rationale for focusing on glycolytic enzymes in our study.

**Fig. 2:**
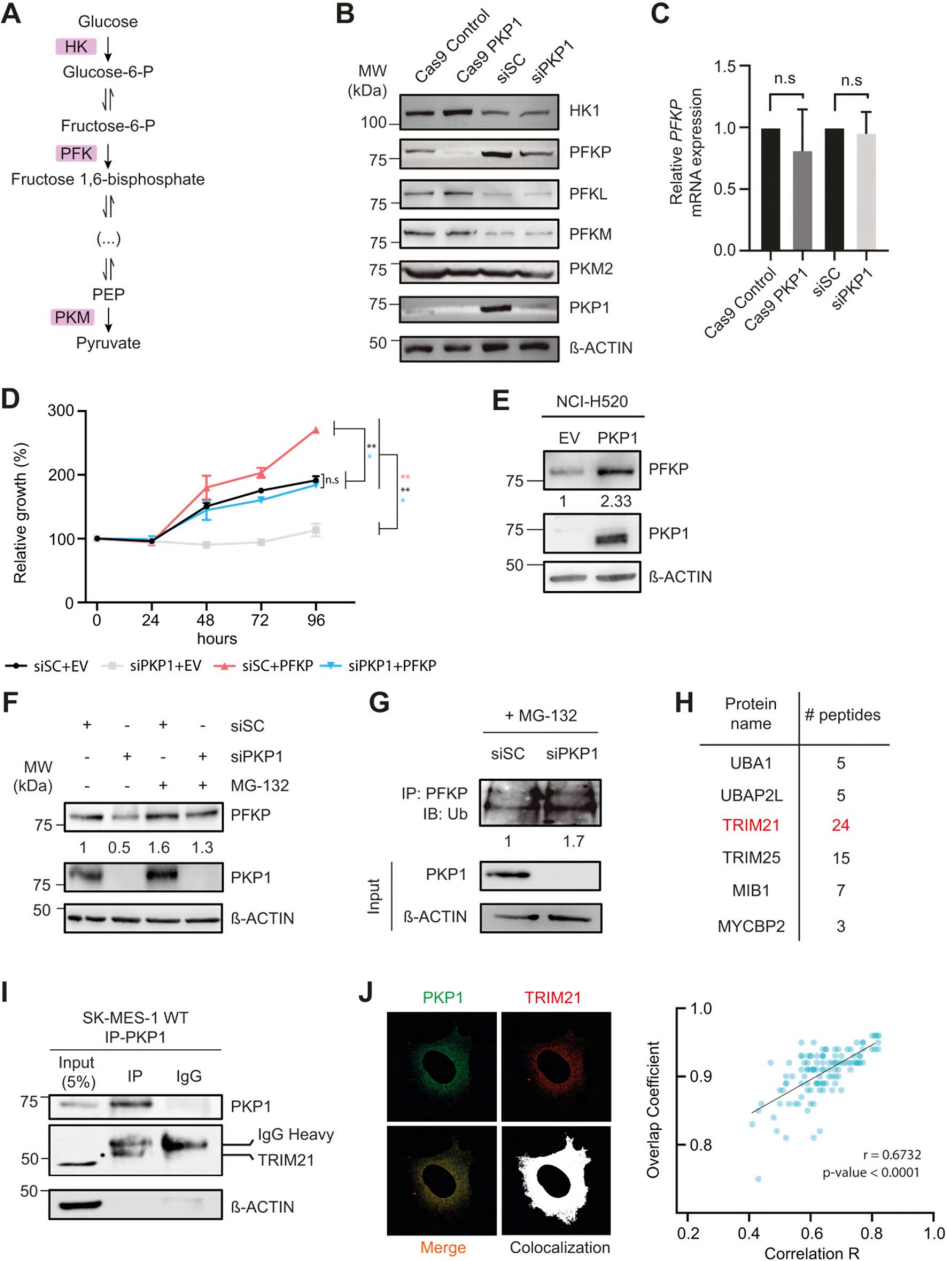
(**A**) Simplified glycolysis diagram highlighting the rate-limiting enzymes: hexokinase (HK), phosphofructokinase (PFK), and pyruvate kinase (PKM). (**B**) Representative immunoblot assay of the glycolytic rate-limiting enzymes: HK1, PFKP, PFKL, PFKM, and PKM2. ß-actin was used as loading control. (**C**) mRNA levels of PFKP in PKP1 KO and KD models. (**D**) Viability assays performed on silenced model of SK-MES-1 cell lines transfected with pCDNA3.1 + EV or pCDNA3.1 + PFKP. Growth was normalized to the EV. *P* values were calculated using an unpaired, two-tailed *t*-test (*t-test P < 0.05; **P < 0.01). (**E**) Immunoblot of PFKP and PKP1 in NCI-H520 overexpression PKP1 model. ß-actin was used as loading control. (**F**) Representative immunoblot of PFKP protein level in the PKP1 KD model upon MG132 treatment. (**G**) PFKP ubiquitination detected by anti-Ub immunoblotting in PKP1 KD model. (**H**) Ubiquitin-related protein interactors identify by IP-MS/MS. (**I**) Immunoblot monitoring co-IP of PKP1 and TRIM21 in SK-MES-1 cell line. ß-actin was used as negative control of the IP. (**J**). Representative image of immunofluorescence of PKP1 and TRIM21 and correlation between Overlap Coefficient and Pearson R (*n* = 117)

These results suggested that PKP1 plays a role in the glycolytic flux. To further investigate the downstream effects, we examined the expression of rate-limiting glycolytic enzymes (Fig. 2.A) upon PKP1 depletion or silencing at both protein (Fig. 2.B and S.3 A) and mRNA levels (Fig. 2.C and S.3B). No changes were observed in mRNA levels overall, but at the protein level, phosphofructokinase platelet (PFKP) levels were reduced upon PKP1 depletion in three different cell lines. PFKP plays a pivotal role as the rate-limiting enzyme catalyzing the conversion of fructose-6-phosphate (F6P) to fructose-1,6-bisphosphate (F1,6BP), and its overexpression has been observed in various tumors, where it correlates with poor prognosis [15]. PFK has two other isoforms, namely muscle (PFKM) and liver (PFKL), but the reduction was exclusive to PFKP (Fig. 2.B).

To assess whether PKP1 promotes lung cancer cell proliferation through PFKP, we performed viability assays in PKP1-depleted cells with ectopic PFKP expression (Fig. 2.D). Silencing of PKP1 (siPKP1 + EV) significantly reduced cell proliferation relative to control cells (siSC + EV). Notably, ectopic expression of PFKP in PKP1-silenced cells (siPKP1 + PFKP) rescued the proliferative capacity to levels comparable to controls, functionally confirming that PFKP mediates the pro-proliferative effect of PKP1. Furthermore, PKP1 overexpression increased PFKP levels by 2.33-fold, demonstrating a functional relationship between both proteins (Fig. 2.E).

These findings prompted us to investigate whether PKP1 could regulate the stability of PFKP. Pretreatment with MG132, a specific 26 S proteasome inhibitor, inhibited the accelerated degradation of PFKP upon PKP1 depletion in different LUSC models (Fig. 2.F and S.5 A). Subsequent ubiquitination assay showed that PKP1 depletion promoted the ubiquitination and degradation of PFKP (Fig. 2.G and S.5B). Together, these results suggest that PKP1 stabilizes PFKP by preventing its proteasomal degradation, thereby enhancing glycolytic activity in LUSC cells.

To identify PKP1-interacting proteins in lung cancer cells, we performed immunoprecipitation (IP) of endogenous PKP1 in SK-MES-1 cells followed by mass spectrometry (MS/MS) analysis. Among the ubiquitin-related interactors, TRIM21, a known E3 ubiquitin ligase targeting PFKP [16], was detected with high confidence (24 unique peptides) (Fig. 2.H). The interaction between PKP1 and TRIM21 was further validated by independent co-IP assays (Fig. 2.I) and confirmed by immunofluorescence (Fig. 2.J), supporting a model where PKP1 binds to TRIM21 and interferes with PFKP ubiquitination, contributing to a high metabolic profile in LUSC cells.

## Supplementary Information

Below is the link to the electronic supplementary material.

Supplementary Material 1

Supplementary Material 2

Supplementary Material 3

Supplementary Material 4

## Data Availability

Raw sequencing data are available at SRA under BioProject accession ID PRJNA1224623.

## Abbreviations

ETC: Electron transport chain
ECAR: Extracellular acidification rate
glycoATP: Glycolytic ATP
glycoPER: Glycolytic Proton Efflux Rate
HK1: Hexokinase 1
IP: Immunoprecipitation
KD: Knockdown
KO: Knockout
LUAD: Lung adenocarcinoma
LUSC: Lung squamous cell carcinoma
mitoATP: Mitochondrial ATP
MRPs: Mitochondrial ribosomal structural proteins
MS/MS: Mass spectrometry/mass spectrometry
NSCLC: Non-small cell lung cancer
OCR: Oxygen consumption rate
OXPHOS: Mitochondrial oxidative phosphorylation
PFK: Phosphofructokinase
PFKL: Phosphofructokinase liver
PFKM: Phosphofructokinase muscle
PFKP: Phosphofructokinase platelet
PKP1: Plakophilin-1
PKM2: Pyruvate kinase
sgRNAs: Single-guide RNAs
SCLC: Small cell lung cancer
siRNA: Small interfering RNA
