# Supplementary material for: PKP1 promotes lung cancer by modulating energy metabolism through stabilization of PFKP
Source: Biomark Res. 2025 Sep 1;13:112. doi: 10.1186/s40364-025-00815-w (PMC12403285; doi:10.1186/s40364-025-00815-w)
Supplement: Supplementary file 1 — Supplementary Material 1 [file 40364_2025_815_MOESM1_ESM.pdf]

# Supplemental Material: PKP1 promotes lung cancer by modulating energy metabolism through stabilization of PFKP

Ritoré-Salazar *et al*

## Table of contents

**SUPPLEMENTAL METHODS** 2

**SUPPLEMENTAL FIGURES** 13

**SUPPLEMENTAL TABLE** 20

**ADDITIONAL FILE DESCRIPTIONS** 21

**REFERENCES** 22

## Supplemental Methods

### Cell culture

Lenti-X 293T (HEK293T) cells were purchased from Takara Bio (#632180). SK-MES-1, HCC95, EPLC-272H and NCI-H520 cells line was obtained from the ATCC collection. SK-MES-1 Cas9 Control and Cas9 PKP1 knockouts cell lines were previously generated [1]. Cell lines were cultured in Dulbecco's Modified Eagle Medium (DMEM) (#L0105-500, Biowest, Riverside, MO, USA) or Roswell Park Memorial Institute (RPMI) 1640, both supplemented with 10% heat-inactivated fetal bovine serum (FBS) (#10270–106, Gibco™ Thermo Fisher Scientific) and 1% penicillin-streptomycin (#P0781-100ML, Sigma-Aldrich) at 37 ºC in 5% $CO_2$. Cells were detached using 1× trypsin-EDTA (#X0930, Biowest, Riverside, MO, USA) and seeded into 96-, 24-, 12-, or 6-well plates.

For PKP1-knockdown experiments, SK-MES-1 cells were transfected with 7.5 nM of a combination of two siRNAs (#s10580 and #s10582, Ambion), using Lipofectamine RNAiMAX reagent (#13778150, Thermo Fisher Scientific) according to the manufacturer's procedure. After 48 h incubation, cells were processed for further analysis. As a control, scramble siRNA (siSC; #4390843, Ambion) was used.

For PFKP-ectopical expression experiments, SK-MES-1 cells were transfected with 1 µg of pcDNA3.1-PFKP (VectorBuilder) using Lipofectamine 2000 reagent (#11668019, Thermo Fisher Scientific), following the manufacturer's instructions. Cells transfected with the empty vector pcDNA3.1-EV were used as control.

Inhibition of proteasome was achieved by treating cells with 10 µM MG-132 (#sc-201270, Santa Cruz) for 12 h. Subsequently, cells were washed with 1× PBS and lysed for either immunoblot or immunoprecipitation assays.

**Lentiviral preparation and titration**

Lentiviral production was performed in Lenti-X 293T cells as previously described [2]. For the titration of the Human Brunello CRISPR knockout pooled library, 100,000 SK-MES-1 cells/well were plated in 6-well plates, and then serial dilutions of the virus ranging from $10^{-2}$ to $10^{-6}$ were prepared. Two days after transduction, puromycin (2 ng/µL) was added to the medium. Cells were then maintained in culture for a period of 2 weeks, after which individual colonies were stained with methylene blue 0.6% in methanol. The number of colonies was then counted, and the colony forming units (CFU) *per* mL of virus were calculated. Generation stable Cas9 expressing parental SK-MES-1, PKP1-KO-1, and PKP1-KO-2 cells were accomplished using LV/Cas9-blast (PMID:25075903) and limiting dilutions selecting populations with 20-30% transduced cells.

**Genome-wide CRISPR/Cas9 knockout screening**

Genome-wide CRISPR/Cas9 knockout screening was performed according to a previously described protocol [3]. In brief, the screen was performed in parental SK-MES-1 cell line and two selected PKP1 knockout clones (PKP1-KO1 and PKP1-KO2). 80 million SK-MES-1 Cas9 Control, KO-1 and KO-2 cells were plated in 20 T-175 flasks per condition. One day after seeding, cells were infected with the Brunello library (multiplicity of infection: MOI = 0.5) and three days later puromycin (2 ng/µL) was added to the growth medium for selection of positively infected cells. Cells were trypsinized, counted, and 80 million cells *per* condition were replated into T-175 flasks every 2-3 days. The puromycin selection was maintained until day 10 after infection, when half the cells (40 million) were pelleted (t = 7 days post puromycin selection), and the rest were kept in culture for further 14 days (until t = 21 days post puromycin selection).

**gDNA extraction and NGS**

Genomic DNA was extracted, the sgRNA expression cassette was amplified by PCR and prepared for targeted next generation sequencing (NGS).

Cell pellets were resuspended in 12 mL of lysis buffer (Tris-HCl 10 mM, EDTA 1 mM, SDS 0.5%, NaCl 150 mM, pH: 10.5) and 60 µL of proteinase K (#539480, Sigma-Aldrich) were added. The lysis reaction was incubated overnight at 55 ºC, and then an ethanol/salt DNA precipitation was performed. Subsequently, the sgRNA expression cassette was amplified by PCR using Ex-Taq® DNA polymerase (#RR01CM, Takara Bio), and primers found in **Additional File 1**. The amplification introduced Nextera adaptors, and the forward primer introduced a stagger region. For each sample 26 PCR reactions with 10 µg of gDNA was performed using the cycling protocol listed below:

| | | | | |
|---|---|---|---|---|
| 95 ºC | 94 ºC | 61 ºC | 72 ºC | 72 ºC |
| 1 min | 30 s | 30 s | 30 s | 10 min |

×26 cycles

Sample were purified using HighPrep™ PCR Clean-Up system (#AC-60050, Magbio), following the manufacturer's recommended protocol. Library preparation and Next generation sequencing was performed by the NGS Core Center, Department of Molecular Medicine, Aarhus University Hospital, Denmark.

**CRISPR screen analysis**

The initial quality check was performed using *FASTQC (v0.11.9)* where the mean sequence quality (Phred score) was above 35 across all samples. Each sample was sequenced to get 50 million reads on average and at minimum 40 million reads. Next, *cutadapt* (v2.10) was used to trim the reads to the sgRNA region with an error rate on 0.1 and a read length between 19 and 21. Then, *bowtie* (v1.3.0) was used to map the reads to all the sgRNAs of the Brunello library using the strict -v 0 -m 1 alignment setting allowing for zero mismatches and only unique alignments. On average, 86% of the reads mapped to a sgRNA and at minimum 84%. The sgRNA coverage was on average 553 and at minimum 452 for each sample.

Finally, a gene essentiality score for every gene was calculated using *JACKS* [4], where a negative gene essentiality score reflects genes were loss of function reduced proliferation. We focused on genes with a negative gene essentiality score across the two PKP1 KO clones and a neutral gene essentiality score in the control cells. JACKS enables joint analysis of all samples to model sgRNA efficiency scores. Here, JACKS was run using a replicate map specifying each sample with the plasmid as control, and the three samples from the PKP1 KO clones at day 21 with the two samples from the parental cell line at day 21 as control. JACKS depletion scores can be found in the **Additional file 2**. Gene Ontology over-representation analysis was performed with WebGestalt [5] applying Bonferroni Hochberg correction as multiple test correction.

**Metabolic assays**

Mitochondrial and glycolytic functions were assessed using a Seahorse Bioscience XFe96 analyzer in combination with the Seahorse Bioscience XF Cell Mito Stress Test, the Bioscience XF ATP Real-Time and the Bioscience XF Cell Glycolytic Rate Test (Agilent Technologies, Santa Clara CA, USA). Briefly, SK-MES-1 were seeded in XFe96 microplates at a density of $1.25 \times 10^4$ cells/well and incubated at 37 ºC overnight. Before the assay, Seahorse XF calibration solution (200 µL/well) was added to a utility plate covered with Hydro Booster and sensor cartridge, then placed overnight in a 37 ºC $CO_2$-free incubator with a hydration probe. The assay medium was prepared with DMEM medium, adding 1 mM pyruvate, 2 mM glutamine, and 10 mM glucose. The cells were washed with assay medium twice and placed in a 37 ºC $CO_2$-free incubator for 1 h. The protocol for determining OCR and ECAR associated with glycolysis was followed. Data analysis was performed using Seahorse Wave software, version 2.6.3. R. All data were normalized to the protein concentration of each well, which was determined using the bicinchoninic acid (BCA) assay (#5000111, BioRad).

**Seahorse XF Cell Mito Stress Test**

OCR was measured using a Seahorse XF Cell Mito Stress Test Kit (#103015-100, Seahorse Bioscience, USA) with the Seahorse XFe96 Extracellular Flux Analyzer following the manufacturer's instructions to evaluate basal respiration, ATP-linked respiration and reserve respiratory capacity. Basal oxygen consumption was assessed following the administration of 2 mM glutamine and 1 mM pyruvate. Mitochondrial ATP production was inhibited using 1 µM oligomycin, which enabled the measurement of mitochondrial oxidative leak. Maximum uncoupling of the mitochondrial electron transport chain was induced by adding 1 µM FCCP. 0.5 µM Rotenone/antimycin A (Rot/AA), inhibitors of complex I and III, were then applied to estimate non-mitochondrial oxygen consumption. To evaluate basal respiration, proton leak, and maximal respiration, the non-mitochondrial respiration values were subtracted.

**Seahorse XF ATP Real-Time Rate Test**

OCR and ECAR assays using Seahorse XF Real-Time ATP Rate Assay Kit (#103092-100, Seahorse Bioscience, USA) are employed to evaluate real-time ATP synthesis rates from both the tricarboxylic acid (TCA) cycle (mitoATP) and glycolysis (glycoATP) using established algorithms. We analyzed. At the same time, we measured proton ($H^+$) efflux and oxygen ($O_2$) consumption rates. By analyzing data collected under basal conditions and after the sequential addition of mitochondrial inhibitors (1 μM oligomycin and 0.5 μM Rot/AA), we determined total cellular ATP production rates and further identified pathway-specific contributions to mitoATP and glycoATP production.

**Seahorse XF Glycolytic Rate Test**

The glycolytic rate assay (#103344-100, Seahorse Bioscience, USA) provides a precise measurement of extracellular acidification specifically caused by glycolysis, eliminating any influence from mitochondrial activity. This methodology allowed for an accurate evaluation of basal glycolytic rates and compensatory glycolysis after the injection of 0.5 μM Rot/AA. The subsequent addition of an inhibitor of hexokinase, the 2-deoxy-D-glucose (2-DG, 50 mM), resulted in the inhibition of glycolytic acidification, leading to a decrease in the Proton Efflux Rate (PER).

**Differential Expression and Functional Enrichment Analysis**

The publicly available transcriptomic dataset **GSE106770** was reanalyzed from the Gene Expression Omnibus (GEO) database. Raw data from the gene expression microarray was processed and analyzed using **R** (version 3.2.3) with the **limma** and **RankProd** packages from the Bioconductor project (https://www.bioconductor.org). Differential expression analysis was performed by comparing cell lines with PKP1 knockdown to control cell lines transfected with a scrambled siRNA. Genes were considered differentially expressed if they showed an adjusted $p$-value < 0.05 and an absolute fold change greater than 1.5. This criteria was used to define both statistically and biologically significant changes.

Functional enrichment analysis was conducted using ShinyGO (version 0.82) [6], selecting pathways from the Kyoto Encyclopedia of Genes and Genomes (KEGG) and Hallmark gene sets from the MSigDB database. Terms with false discovery rate (FDR) < 0.05 were considered significantly enriched.

**Viability assays**

$2.5 \times 10^5$ SK-MES-1 cells *per* well were seeded in 6-well plates (day 0). On day 1, cells were transfected with 7.5 nM siRNA targeting *PKP1* (siPKP1) or a non-targeting siRNA control (siSC). On day 2, cells were transfected with either a PFKP overexpression plasmid or an empty vector. This generated four experimental conditions: siSC + EV, siSC + PFKP, siPKP1 + EV, and siPKP1 + PFKP. On day 3, cells were trypsinized, counted, and re-seeded at a density of $5 \times 10^3$ cells *per* well into 96-well plates in triplicate for each condition and time point. Cell viability was assessed at 24-hour intervals using a resazurin-based assay. Briefly, 20 µL of resazurin sodium salt (# R7017, Sigma-Aldrich) diluted 1:200 from a 0.1 M stock solution in PBS was added directly to each well and incubated for 4 h at 37 °C in the dark. Fluorescence (excitation: 560 nm, emission: 590 nm) was measured using a GloMax Discover Microplate Reader (Promega).

**Stable PKP1 Expression**

Stable expression of exogenous PKP1 was achieved through lentiviral transduction. Lentiviral particles were generated by transient co-transfection of HEK293T cells using LipoD293 (SignaGen, SL100668) according to the manufacturer's protocol. A 2:1:3 ratio of plasmids was used, including the packaging plasmid psPAX.2 (Addgene #12260), the envelope plasmid encoding VSV-G (Addgene #8454), and either pLVX-PKP1A-Myc-DDK-IRES-ZsGreen1 or the control empty vector pLVX-IRES-ZsGreen1 (Clontech, 632187). Viral supernatants were collected at 48 and 72 hours post-transfection, filtered through 0.45 µm membranes, and stored at −80 °C. NCI-H520 cells at ~80% confluence were subjected to three rounds of infection in the presence of 8 µg/mL polybrene. Following transduction, cells were analyzed

by flow cytometry using a BD FACSCanto II (BD Biosciences), and, when necessary, sorted using a BD FACSAria (BD Biosciences) for downstream applications.

**Immunofluorescence preparations**

A total of 5 × $10^4$ SK-MES-1 cells/well were seeded in cover-slides and incubated for 24 h at 37 °C in the presence of 5% $CO_2$. The following day seeded cells were fixed with 4% paraformaldehyde in 1× PBS for 7 min at room temperature and permeabilized with 0.2% Triton X-100 in 1 × PBS for 5 min. Blocking was performed using 3% BSA in PBS for 1 h at room temperature. Cells were then incubated overnight at 4 °C in a humidified chamber with the following primary antibodies diluted in blocking solution: anti-PKP1 (#HPA027221, Sigma Aldrich, 1:150) and anti-TRIM21 (#92043, Cell Signaling, 1:100). After washing, cells were incubated for 1 h at room temperature with the corresponding secondary antibodies: Alexa Fluor 488-conjugated antibody (#A-11008, ThermoFisher) for PKP1 detection and Alexa Fluor 647-conjugated antibody (#A-21244, ThermoFisher) for TRIM21 detection. Nuclei were counterstained with 5 ng/µL 4,6-diamidino-2-phenylindole (DAPI) in PBS for 5 min. Finally, samples were mounted on microscope slides using mounting medium (Mowiol®) and allowed to dry at room temperature.

Images were acquired using a Zeiss LSM-710 confocal microscope equipped with appropriate laser lines and emission filters for multichannel fluorescence detection. Cytoplasmic colocalization analyses were performed using the colocalization module of the Zeiss ZEN software (Carl Zeiss, Germany). A total of 117 cells were manually selected and analyzed across multiple fields. For each pair of fluorescent channels, the software computed two quantitative parameters: the Overlap Coefficient, which indicates the proportion of spatial overlap between signals regardless of their intensity correlation (ranging from 0 to 1), and the Pearson's Correlation Coefficient (R), which quantifies the linear relationship between fluorescence intensities of the two channels at each pixel (ranging from –1 to +1). To assess the internal consistency of the colocalization parameters, we performed a correlation analysis

between the Overlap Coefficient and the Pearson's Correlation Coefficient (R) obtained for each cell. This analysis was conducted using GraphPad Prism v.9 and statistical significance was set at $p < 0.05$. Raw data is provided in **Additional File 3**.

**Protein extraction and Immunoblot**

Cell lysates were prepared in RIPA buffer (150 mM NaCl, 1% NP-40, 0.5% sodium deoxycholate, 0.1% SDS and 50 mM Tris-HCl pH: 7.5) containing protease and phosphatase inhibitors (0.2 mM PMSF, 7 mM $VO_4$ and 1× complete Mini EDTA-free Protease Inhibitor Cocktail Tablets). Protein concentration was determined by Bradford (#A6932, PanReac AppliChem) using BSA as a standard. 40 µg total protein was subjected to 8-12% SDS-PAGE. The resolved samples were transferred onto PVDF membranes and blocked for 1 h in 1× PBS containing 5% not-fat dry milk and 0.1% Tween (#0777, Ambresco). Then, they were incubated overnight at 4 ºC with primary antibodies (**anti-PKP1**, #HPA027221, Sigma-Aldrich, 1:250; **anti-HK1**, #2024, Cell Signaling, 1:1000; **anti-PKM1/2**, #3190, RatBiotech, 1:1000; **anti-PFKP**, #8164, Cell Signaling, 1:1000, **anti-PFKM**, #sc-377246, Santa Cruz, 1:500; **anti-PFKL**, #sc-393713, 1:500; **anti-Ubiquitin**, #3936, Cell Signaling, 1:1000; **anti-TOM20**, #42406, Cell Signaling, 1:1000; **anti-TRIM21**, #92043, Cell Signaling, 1:1000); **anti-β-actine**, #3700, Cell Signaling, 1:10000), followed by an incubation with the corresponding secondary antibodies (**anti-rabbit HRP**, Cat#P0448, Dako, 1:2000 or **anti-mouse HRP**, Cat#P0447, Dako, 1:1000) at room temperature for 1 h. The target protein bands were visualized using Clarity Western ECL Substrate and ImageQuant LAS4000 (GE Healthcare). Protein bands were quantified using ImageJ v1.53a software and normalized according to their respective β-actin levels.

**Real-time quantitative polymerase chain reaction**

Real-time quantitative PCR (RT-qPCR) was optimized using the Applied Biosystems 7900HT Real-Time PCR System with cDNA prepared after a reverse transcription of 1 µg total RNA (#K1691, RevertAid RT Kit, Thermo Fisher Scientific). All qPCR reactions followed the KAPA SYBR® FAST qPCR Master Mix (#KK4600, Merck) recommendations. Relative expression was calculated using *β-ACTIN* as a housekeeping gene and applying the DDCt method. Primers for each gene are shown in **Table Supplementary 1**. All experiments were carried out in biological triplicates.

**Co-immunoprecipitation**

Co-immunopreciptation of PFKP from SK-MES-1 siSC and siPKP1 cell lysates and PKP1 from SK-MES-1 was performed using Dynabeads$^{TM}$ Protein G (#10004D, Thermo Fisher Scientific). For PFKP or PKP1 immunoprecipitacion magnetic beads (25 µL/reaction) were pre-coupled with anti-PFKP antibody (#3936, Cell Signaling, 1:50) or anti-PKP1 antibody (#HPA027221, Sigma-Aldrich, 1:20), respectively, and their corresponding IgG control at the same concentration. The coupling reaction was set for 1 hour at room temperature in a rotating wheel, with a final volume of 250 µL completed with RIPA buffer containing protease inhibitors. After coupling, tubes were placed in a magnetic rack and the supernatant was removed. Subsequently, 500 µg of protein *per* reaction were incubated overnight on the rotating wheel with the antibody-magnetic bead complexes. The following day, the supernatant was removed, beads were washed three times with 1× PBS while rotating and finally resuspended in 25 µL of loading buffer. Samples were subjected to immunoblot analysis to assess PFKP ubiquitination, PKP1–protein interaction and MS/MS analysis.

**Mass Spectrometry**

PKP1 immunoprecipitation was performed first, followed by sample preparation, digestion, and separation using UPLC chromatography. Mass spectrometry analysis (MS/MS) was carried out on a Thermo Orbitrap Fusion mass spectrometer (Q-OT-qIT, Thermo Fisher Scientific) according to standardized protocols. Raw data were processed using Proteome Discoverer software (version 2.1.0.81, Thermo Fisher Scientific). Finally, a filtered list of proteins was functionally analyzed in our laboratory using the clustering tool available in DAVID v2024q4.

**Statistical analyses**

The results are expressed as the average $\pm$ standard deviation (SD) and represent at least three biological replicates. Statistical differences were analyzed using unpaired, two-tailed t-tests or MANOVA test. Significance was considered when $p < 0.05$.

## Supplemental Figures

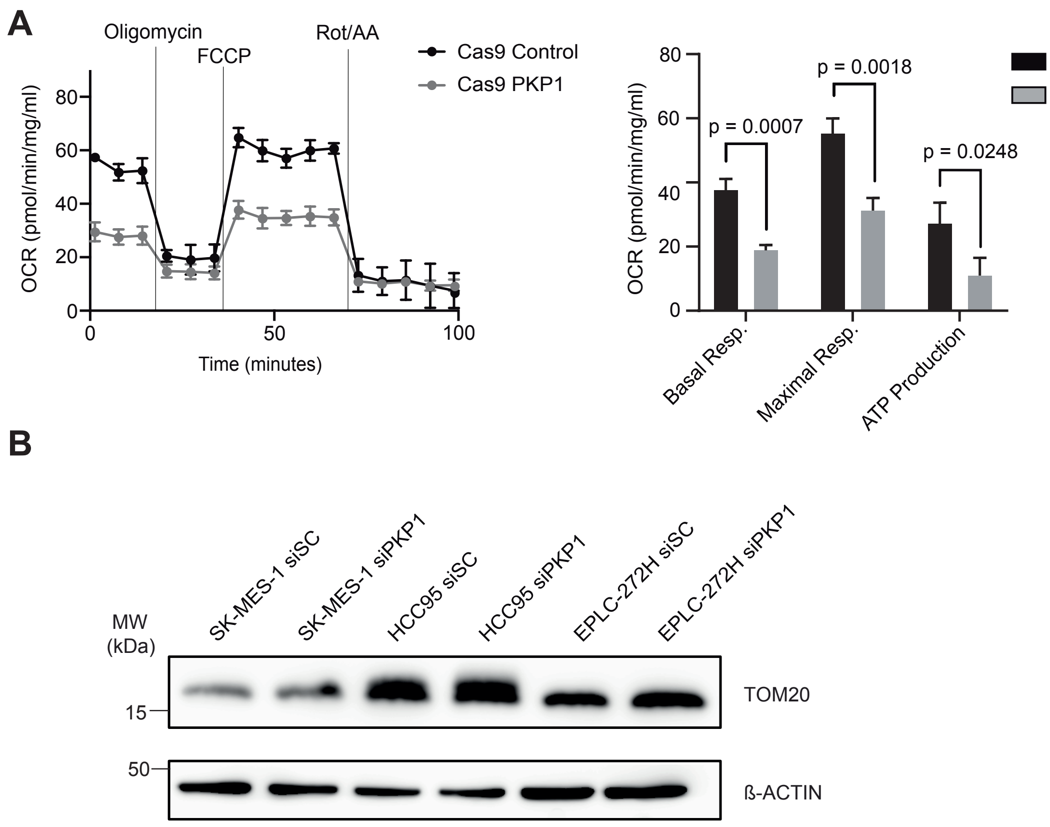


**Figure S1.** (**A**) **Left panel**: OCR profile at baseline and in response to oligomycin, carbonyl cyanide 4-(trifluoromethoxy) phenylhydrazone (FCCP), and antimycin A plus rotenone (Rot/AA) of SK-MES-1 Cas9 Control and Cas9 PKP1. **Right panel**: Bar plots show the quantification of basal respiration, maximal respiration and ATP production in the KO model. (**B**) Immunoblot assay of the mitochondrial marker TOM20 in SK-MES-1, HCC95 and EPLC-272H silenced model. β-actin was used as loading control.

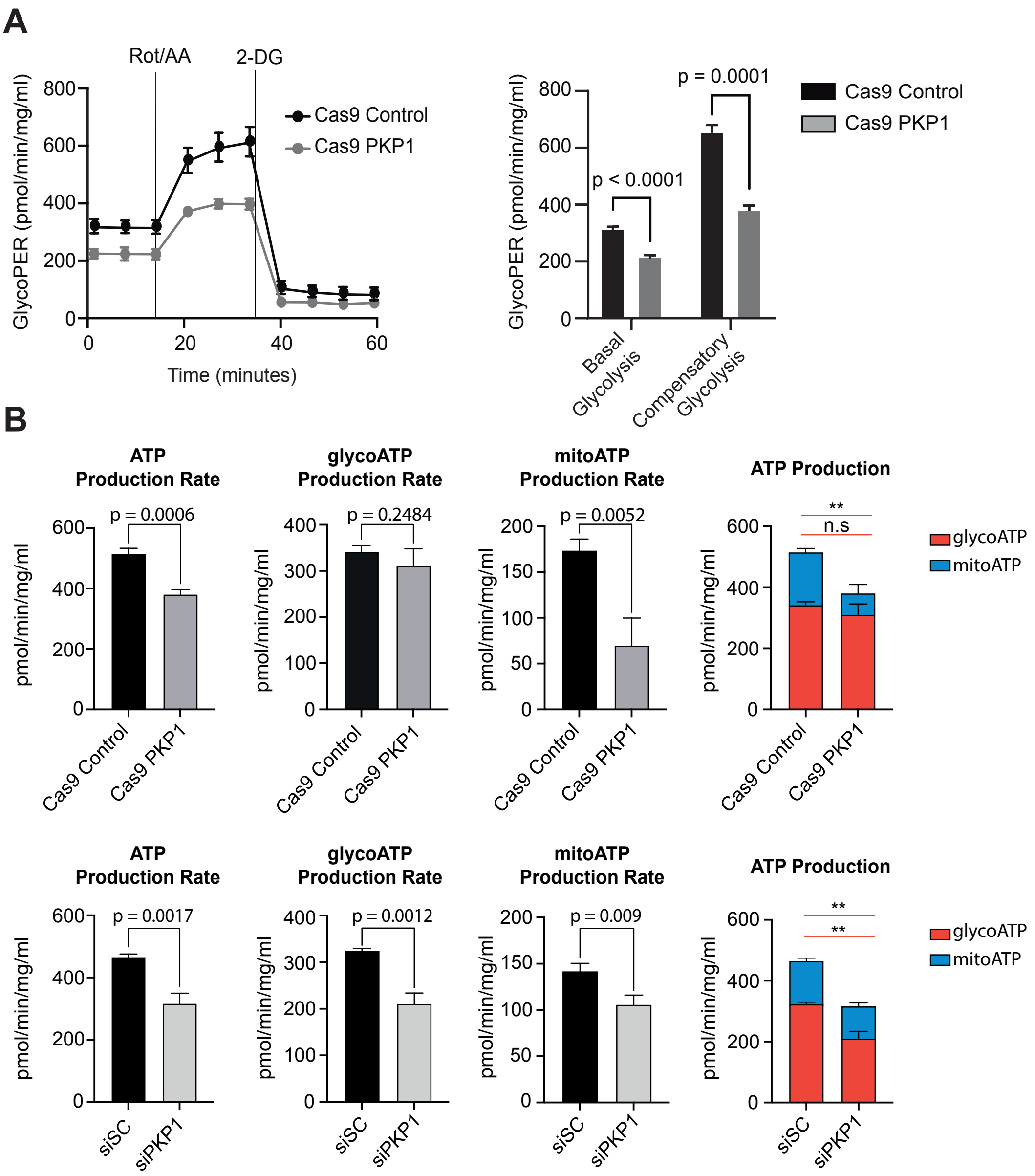


**Figure S2**. (A) **Left panel**: GlycoPER profile at baseline and in response to Rot/AA and 2-deoxy-D-glucose (2-DG) of SK-MES-1 Cas9 Control and Cas9 PKP1. **Right panel**: Bar plots show quantification of basal and compensatory glycolysis in the KO model. (**B**). Bar plots show quantification of total ATP production, ATP produced via glycolysis and via mitochondria in both the KO and silenced models. Results were normalized to total concentration (mg/ml) of protein of each well. All experiments were conducted in triplicates and *P* values were calculated using an unpaired, two-tailed *t*-test (**t-test P < 0.05; **P < 0.01).*

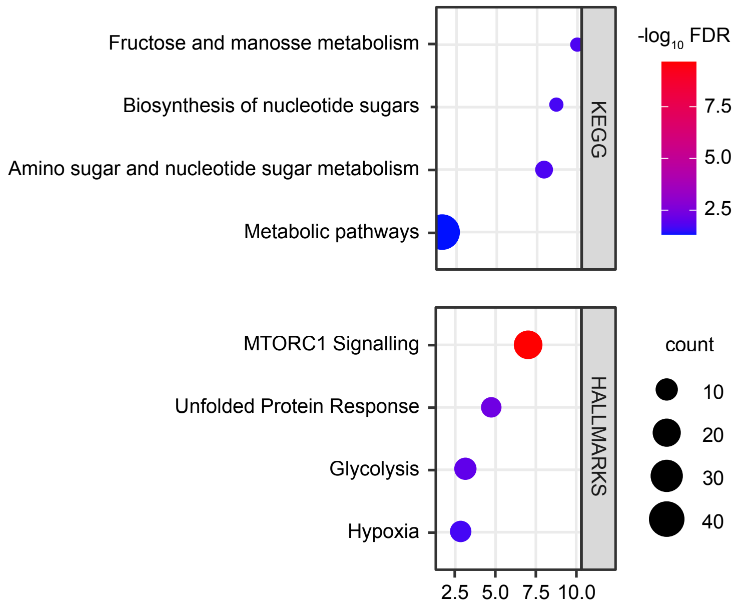


**Figure S3.** Functional enrichment analysis of differentially expressed genes following *PKP1* knockdown by siRNA. Significantly enriched terms from the KEGG (top) and HALLMARK (bottom) databases were identified using ShinyGO (v0.82). Dot size indicates the number of genes associated with each term ("count"), while color represents statistical significance based on the $-\log_{10}$ (FDR), as shown in the color scale.

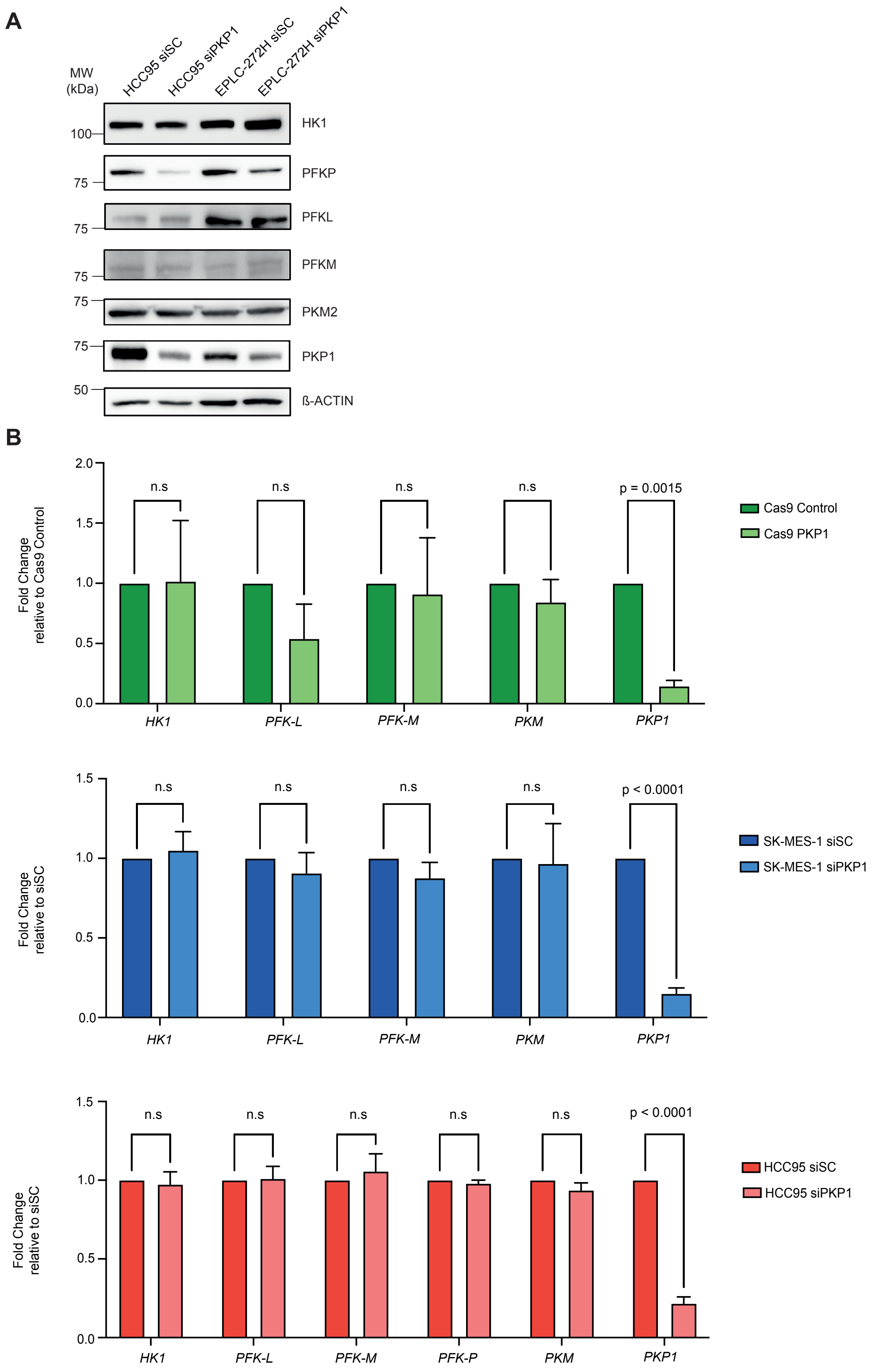


(See legend on next page.)

**Figure S4.** (**A**) Immunoblot assay of the glycolytic rate-limiting enzymes: HK1, PFKP, PFKL, PFKM, PKM2 in HCC95 and EPLC-272H silenced model. β-actin was used as loading control. (**B**) mRNA levels of glycolytic rate-limiting enzymes in PKP1 knockout and knockdown (SK-MES-1 and HCC95) models. *PKP1* was used as positive control. All experiment were performed in triplicates and *P* values were calculated using an unpaired, two-tailed *t*-test.

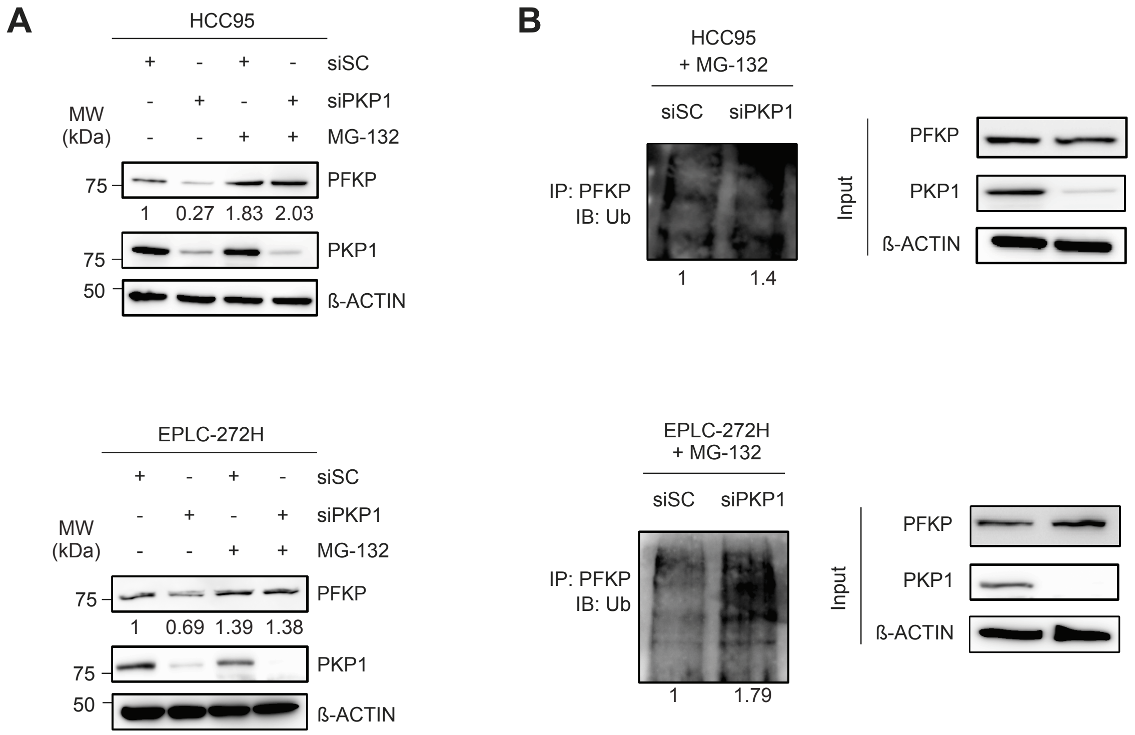


**Figure S5.** (**A**) Representatives immunoblot of PFKP protein level in HCC95 and EPLC-272 silenced model upon MG132 treatment. (**B**) PFKP ubiquitination detected by anti-Ub immunoblotting in PKP1 both silenced models.

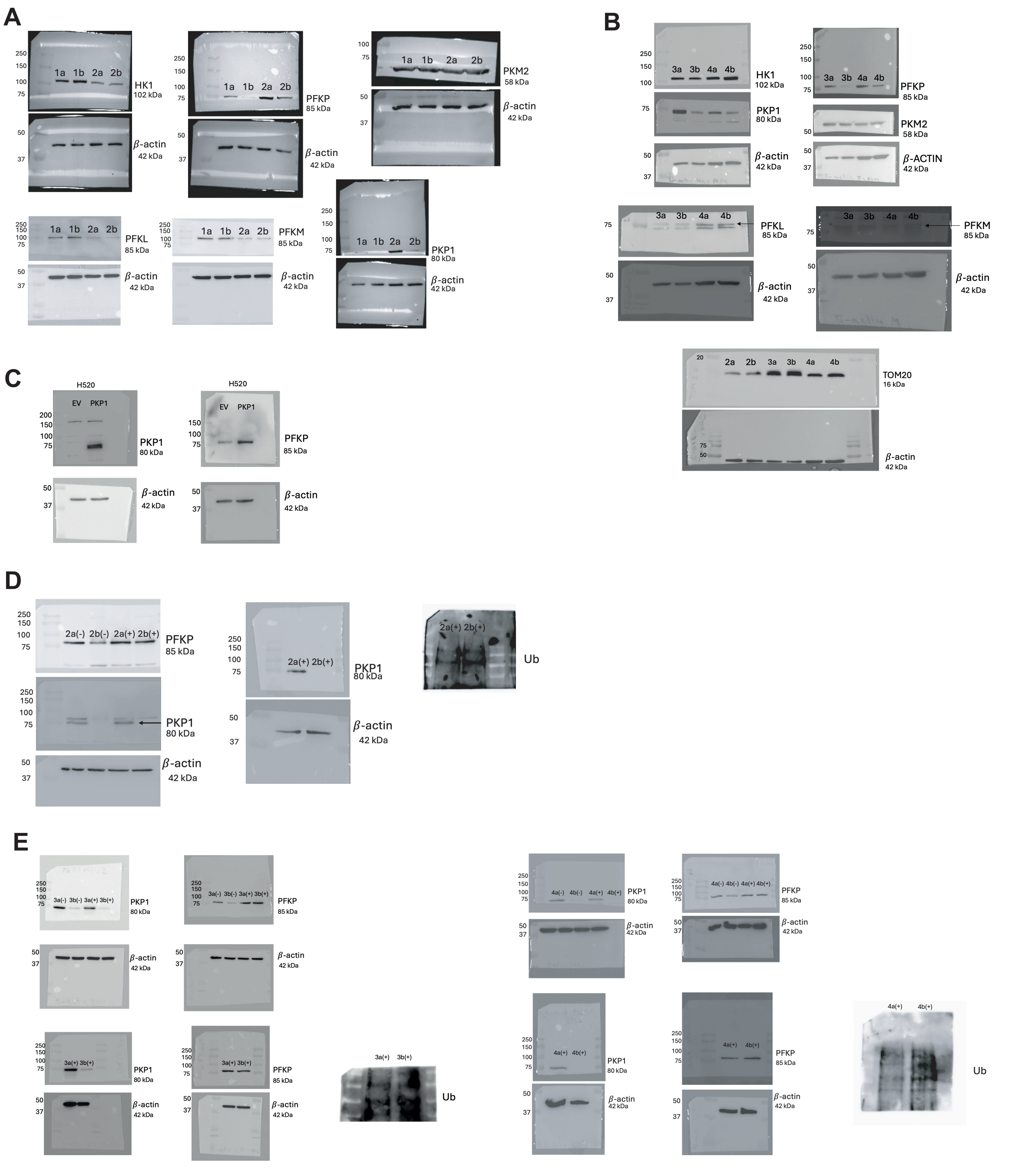


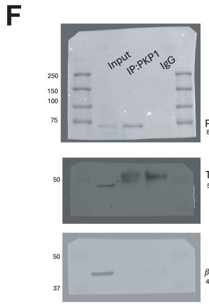


**Legend:**

1a. SK-MES-1 Cas9 Control
1b. SK-MES-1 Cas9 PKP1
2a. SK-MES-1siSC
 2a(-). Untreated
 2a(+). MG-132
2b. SK-MES-1siPKP1
 2b(-). Untreated
 2b(+). MG-132
3a. HCC95 siSC
 3a(-). Untreated
 3a(+). MG-132
3b. HCC95 siPKP1
 3b(-). Untreated
 3b(+). MG-132
4a. EPLC-272H siSC
 4a(-). Untreated
 4a(+). MG-132
4b. EPLC-272H siSC
 4b(-). Untreated
 4b(+). MG-132

**Figure S6.** Uncropped blots of Fig. 2.B (A); Fig. S4.A (B); Fig 2.E (C); Fig 2.F-G (D); Fig. S5-A-B (E). Fig. 2.I (F).

## Supplemental Table

**Table 1.** List of oligonucleotides used for RT-qPCR in this study.

| **Gene** | **Forward Sequence (5' to 3')** | **Reverse Sequence (5' to 3')** |
|---|---|---|
| *PKP1* | TCAGCAACAAGAGCGACAAG | TCAGGTAGGTGCGGATGG |
| *HK1* | CCCTAAATGCTGGGAAACAAAG | GTCTTCAGCGTCTCAGAGATC |
| *PFKP* | AGGCAGTCATCGCCTTGCTAGA | ATCGCCTTCTGCACATCCTGAG |
| *PFKL* | AAGAAGTAGGCTGGCACGACGT | GCGGATGTTCTCCACAATGGAC |
| *PFKM* | GCTTCTAGCTCATGTCAGACCC | CCAATCCTCACAGTGGAGCGAA |
| *PKM2* | GGGAGAGAAGGGAAAGAACATC | TCTCTGCAGGAATCTCAATGC |
| *β-ACTIN* | GGCGGCACCACCATGTACCCT | AGGGGCCGGACTCGTCATACT |

## Additional File Descriptions

**Additional File 1.** Description of NGS primers.

**Additional File 2.** Joint Analysis of CRISPR/Cas9 knockout screens (JACKS) depletion scores. Columns show gene names, knockout scores (KO_score) and control scores (Control_score). Negative values indicate a higher sensitivity or dependency of the gene in the respective condition.

**Additional File 3.** Immunofluorescence raw data.

## References

1. Martin-Padron J, Boyero L, Rodriguez MI, Andrades A, Díaz-Cano I, Peinado P, et al. Plakophilin 1 enhances MYC translation, promoting squamous cell lung cancer. Oncogene. 2020 Dec 10;39(32):5479–93.
2. Ryø LB, Thomsen EA, Mikkelsen JG. Production and Validation of Lentiviral Vectors for CRISPR/Cas9 Delivery. Methods Mol Biol. 2019;1961:93-109.
3. Thomsen EA, Mikkelsen JG. CRISPR-Based Lentiviral Knockout Libraries for Functional Genomic Screening and Identification of Phenotype-Related Genes. Methods in Molecular Biology. 2019;1961:343–57.
4. Allen F, Behan F, Khodak A, Iorio F, Yusa K, Garnett M, et al. JACKS: joint analysis of CRISPR/Cas9 knockout screens. Genome Res. 2019 Mar 1;29(3):464–71.
5. Wang J, Duncan D, Shi Z, Zhang B. WEB-based GEne SeT AnaLysis Toolkit (WebGestalt): update 2013. Nucleic Acids Res. 2013 Jul 1;41(W1):W77–83.
6. Ge, S. X., Jung, D., & Yao, R. ShinyGO: a graphical gene-set enrichment tool for animals and plants. Bioinformatics. 2020. 36(8), 2628–2629.
