# Supplementary figures and images for: PKP1 promotes lung cancer by modulating energy metabolism through stabilization of PFKP
Source: Biomark Res. 2025 Sep 1;13:112. doi: 10.1186/s40364-025-00815-w (PMC12403285; doi:10.1186/s40364-025-00815-w)

**#1-11**


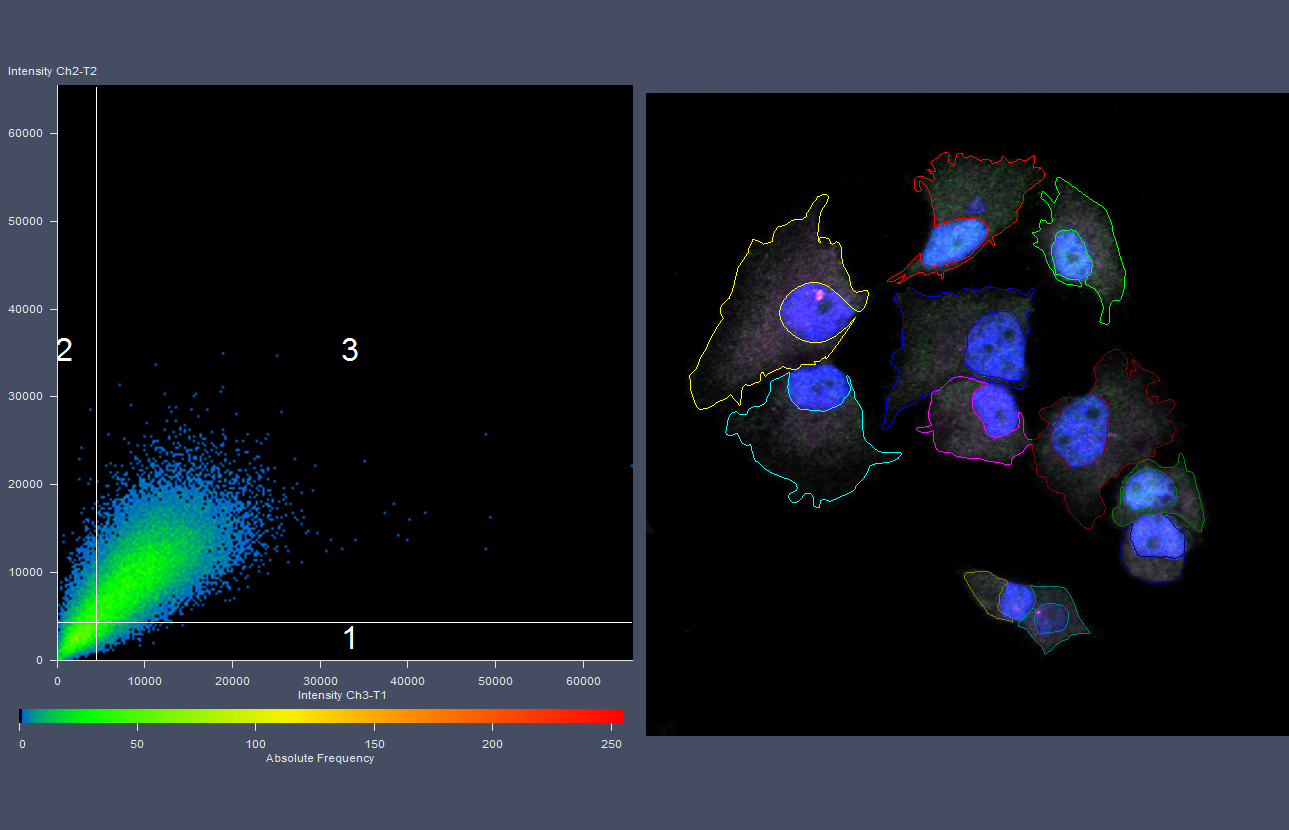


**#12-19**


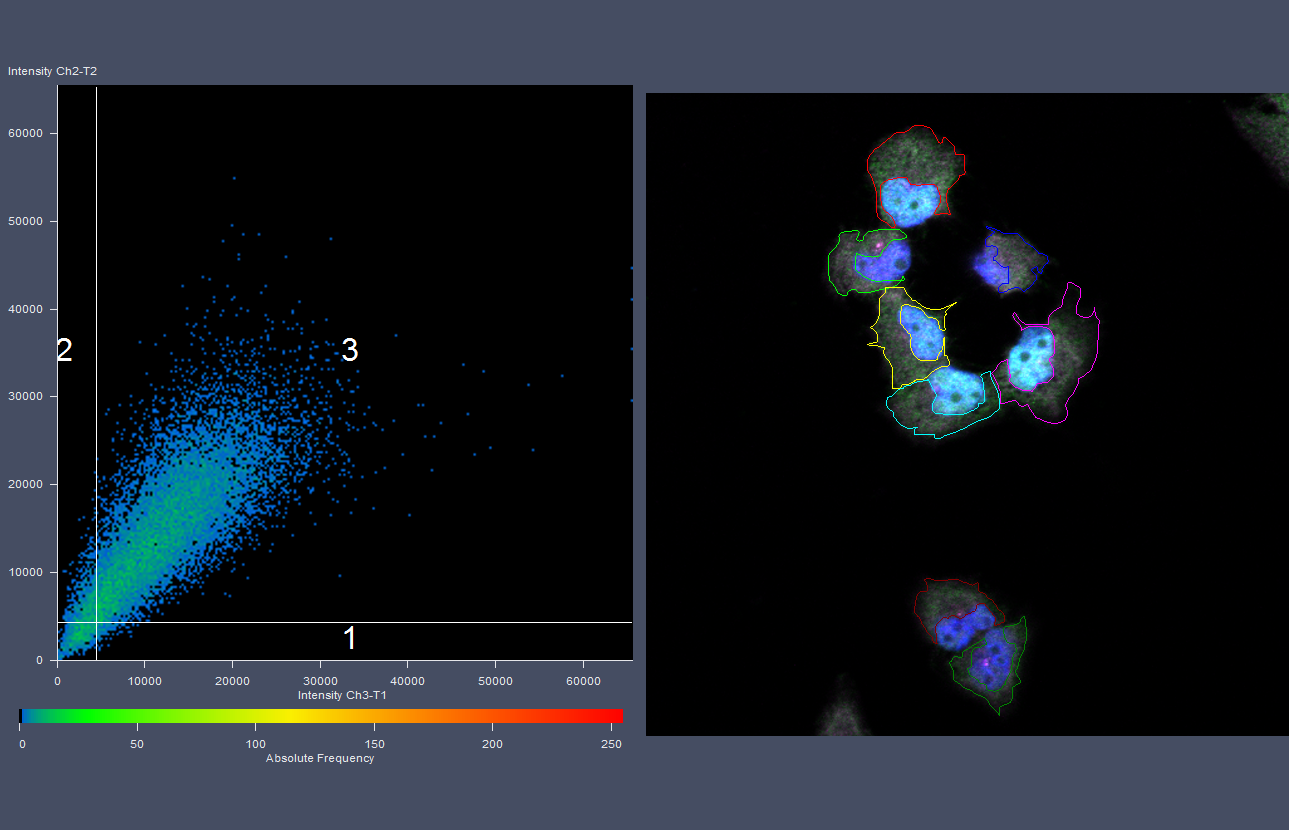


**#20-26
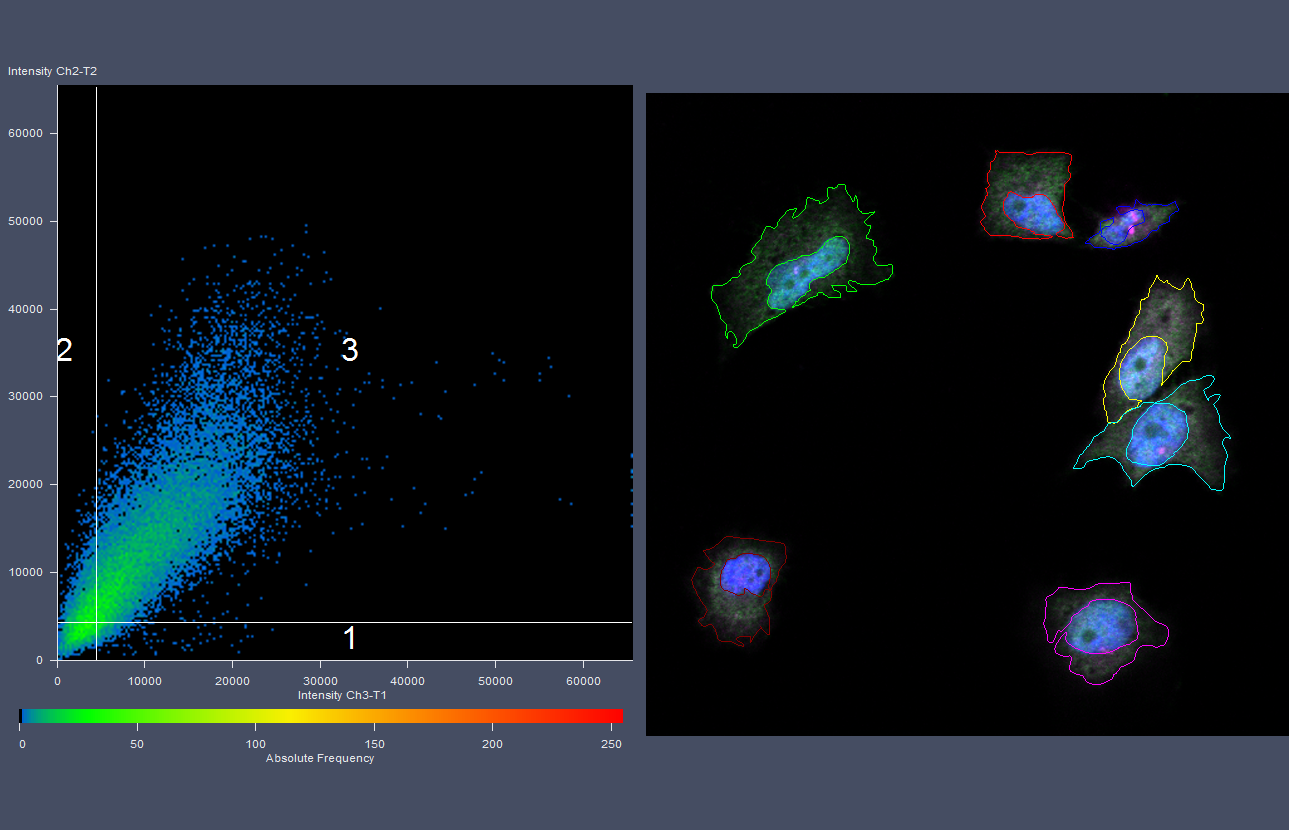
**

**#27-34
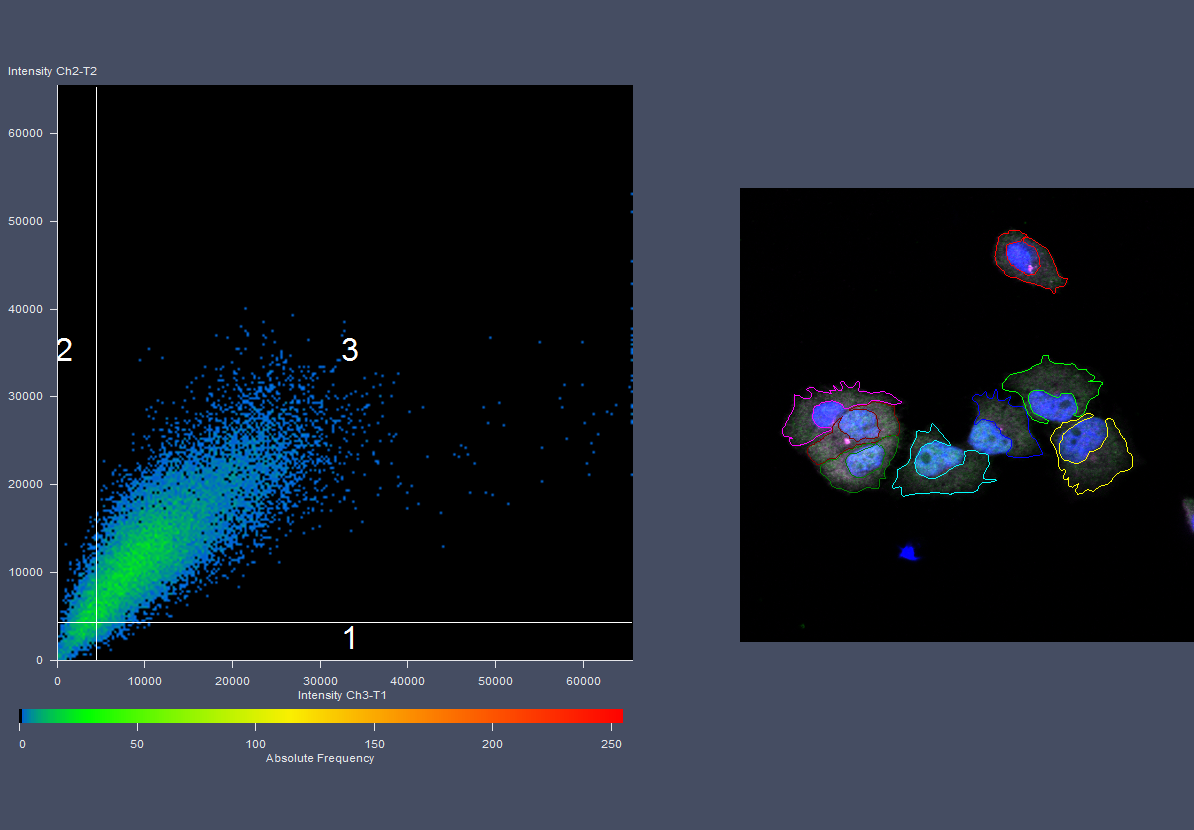
**

**#35-45
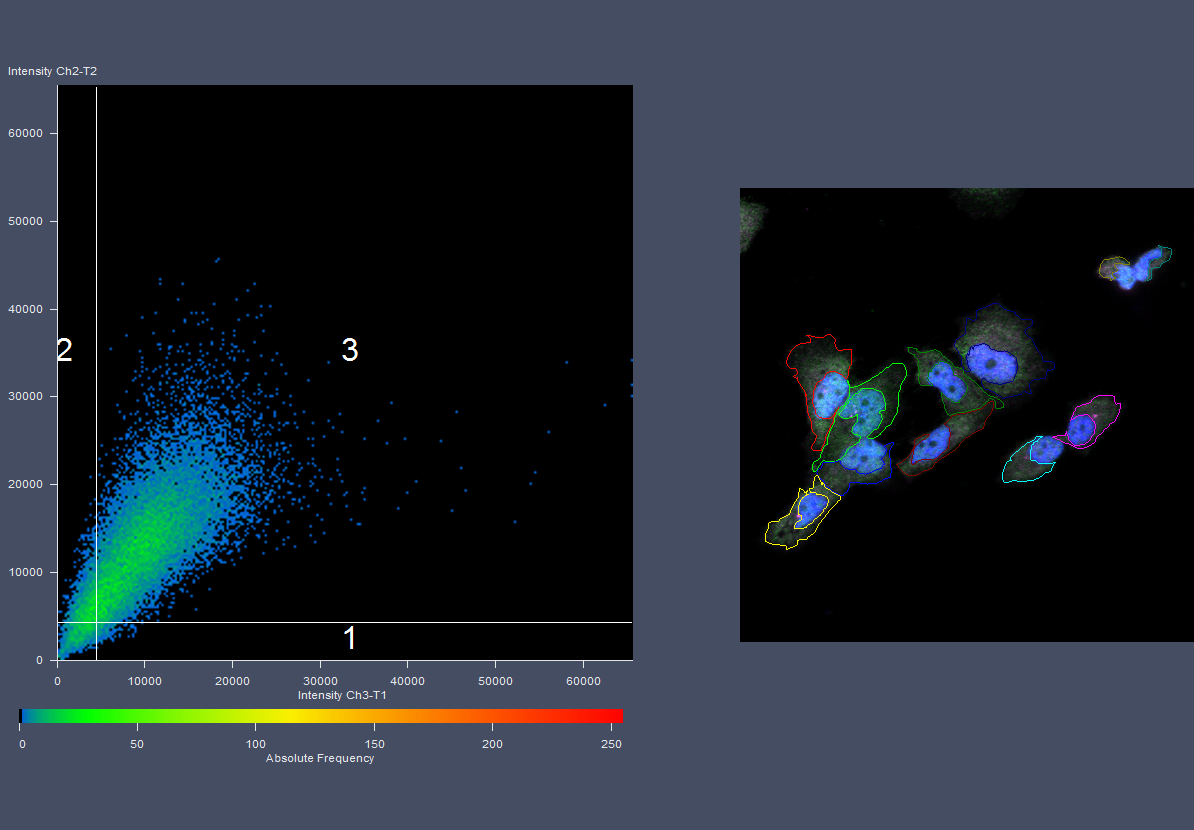
**

**#46-59
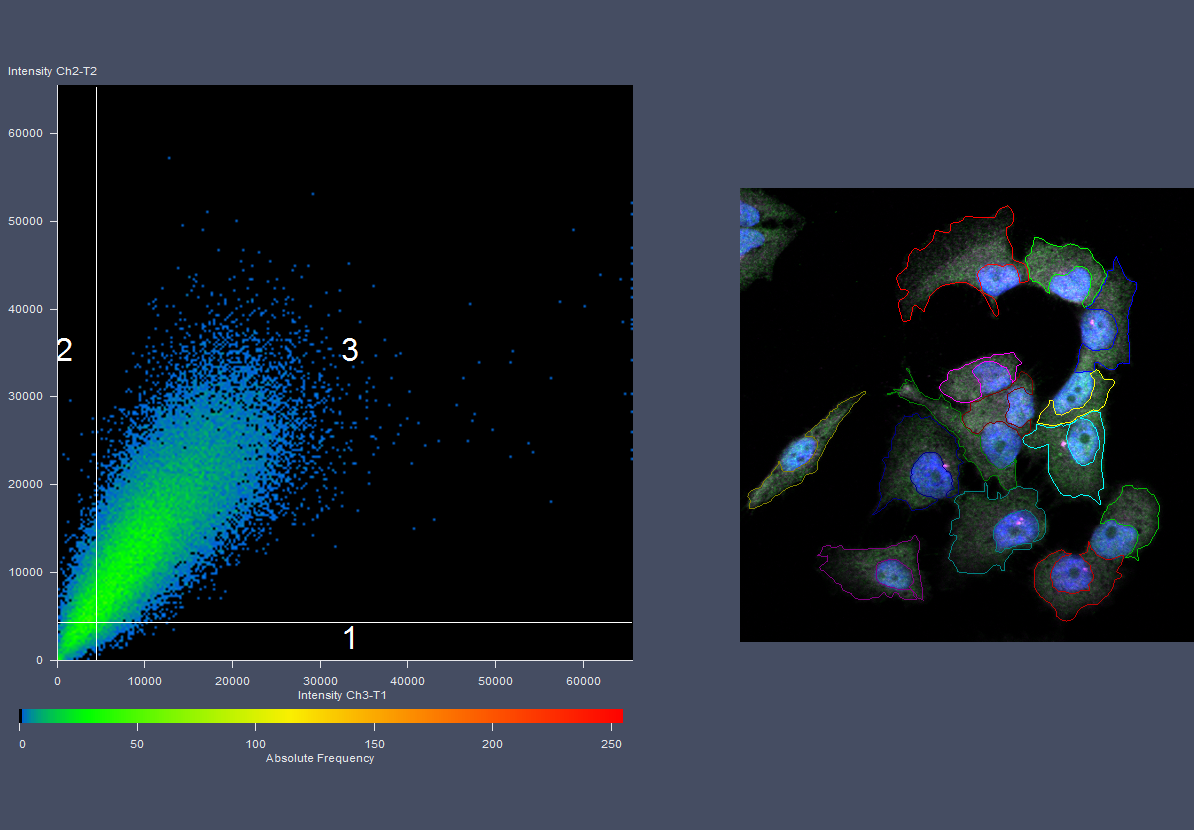
**

**#60-69
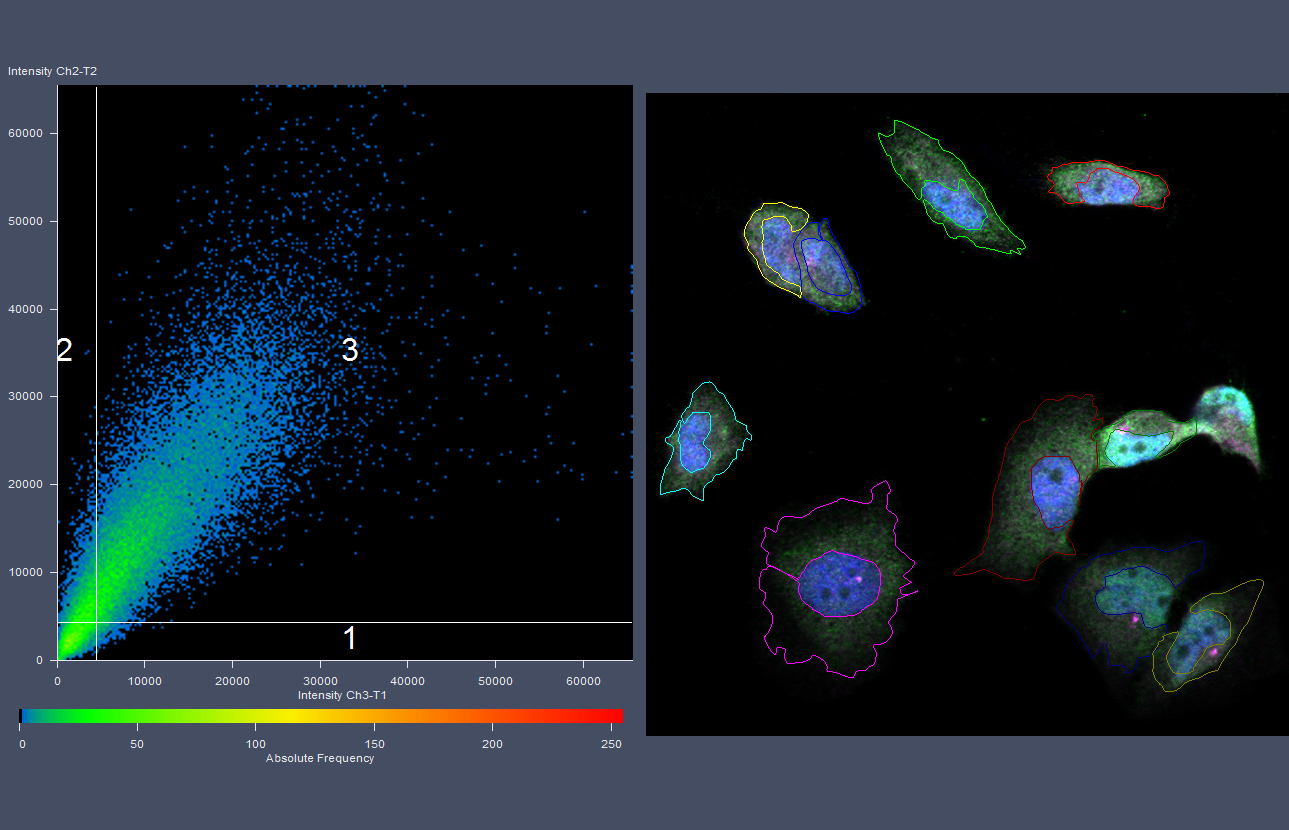
**

**#70-82
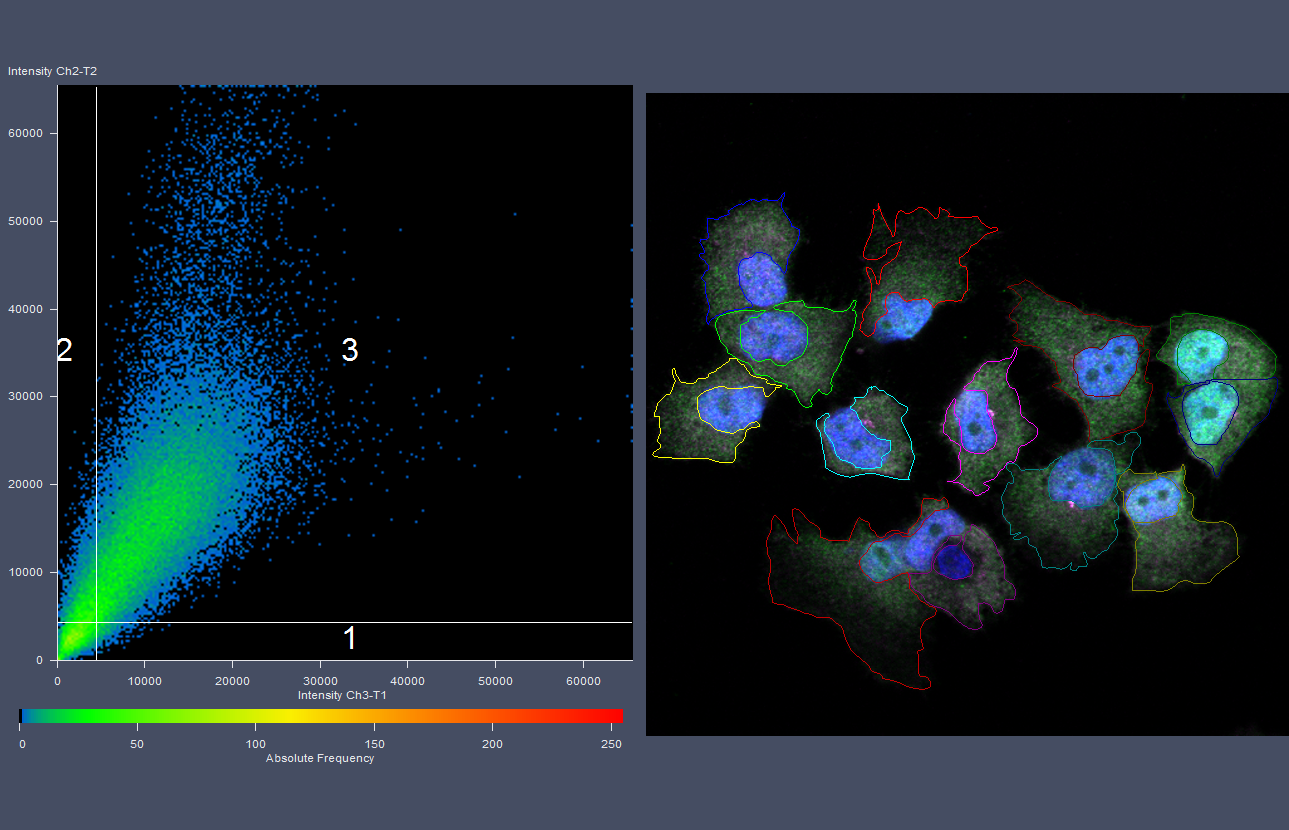
**

**#83-94
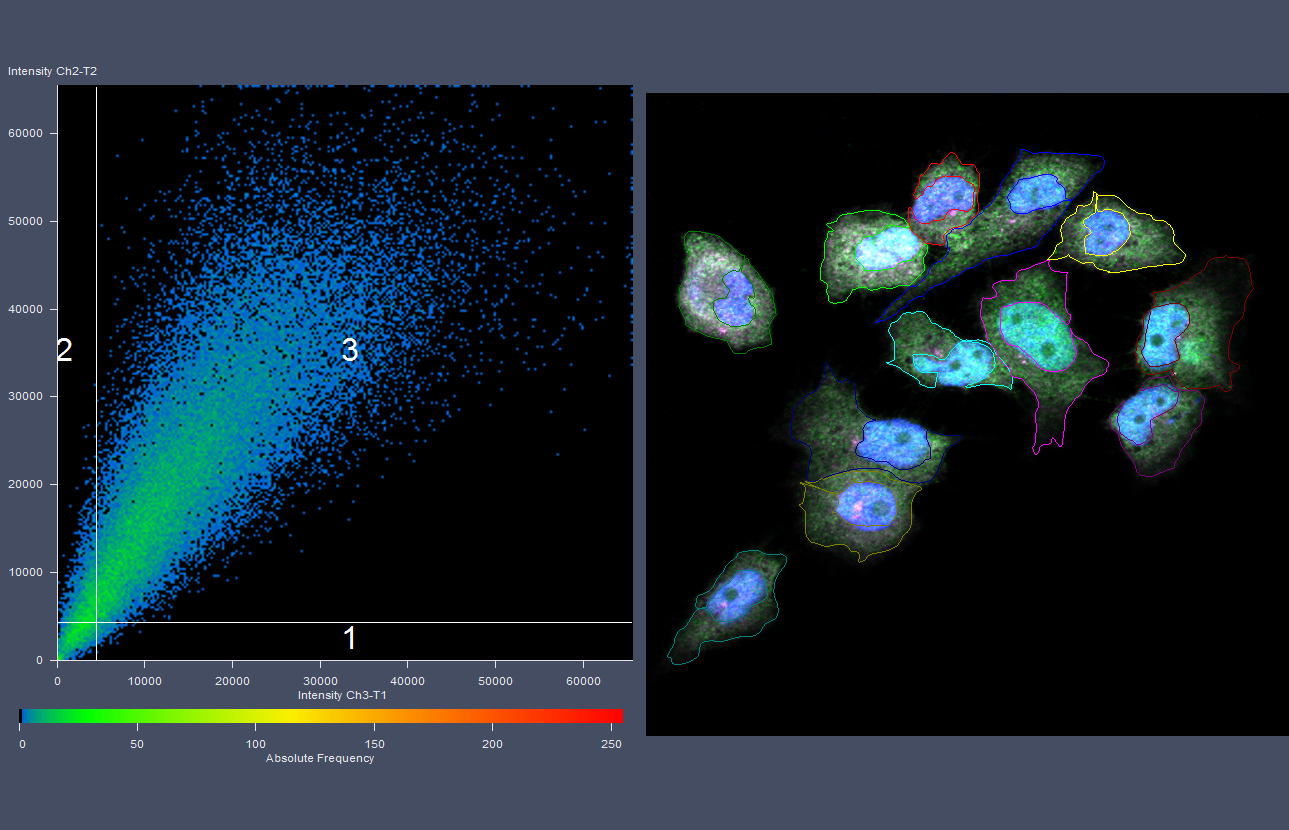
**

**#95-104**

**
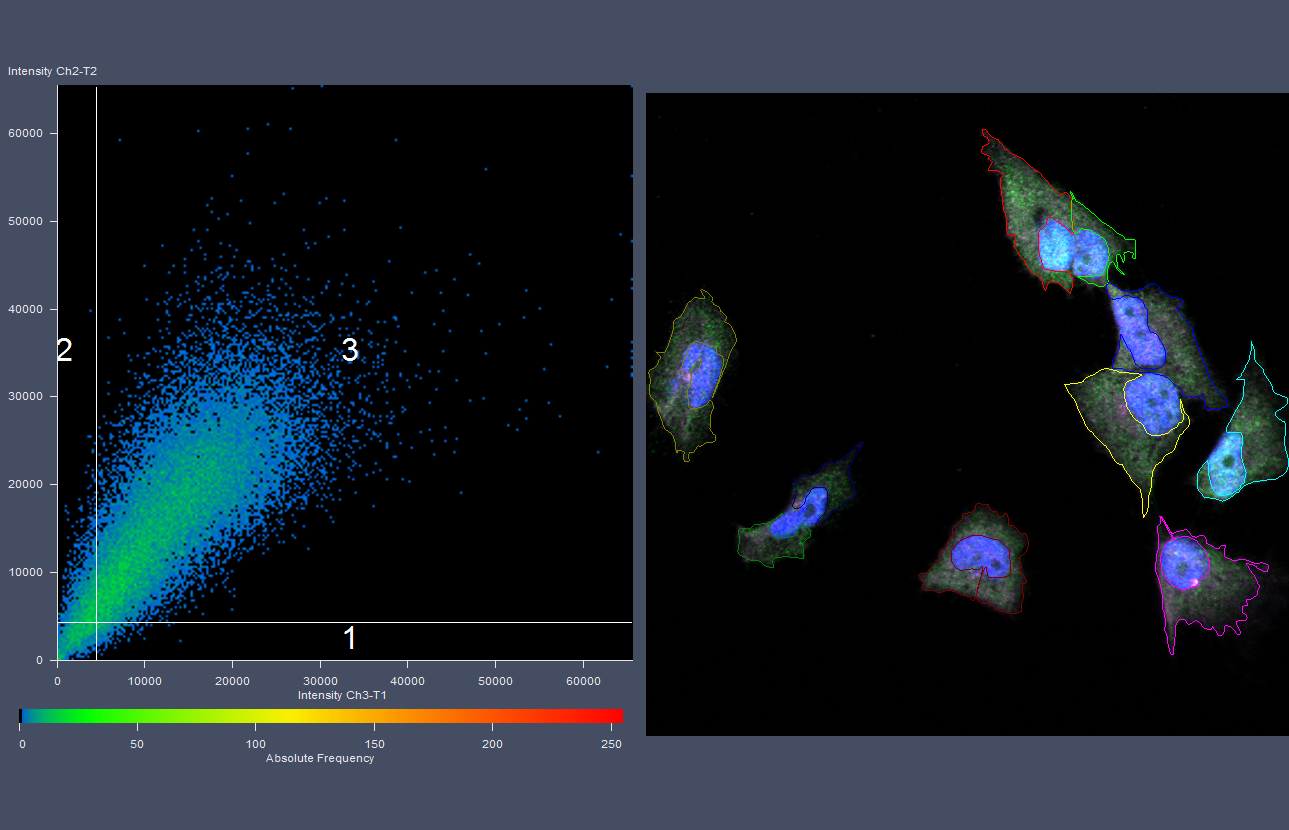
**

**#105-112
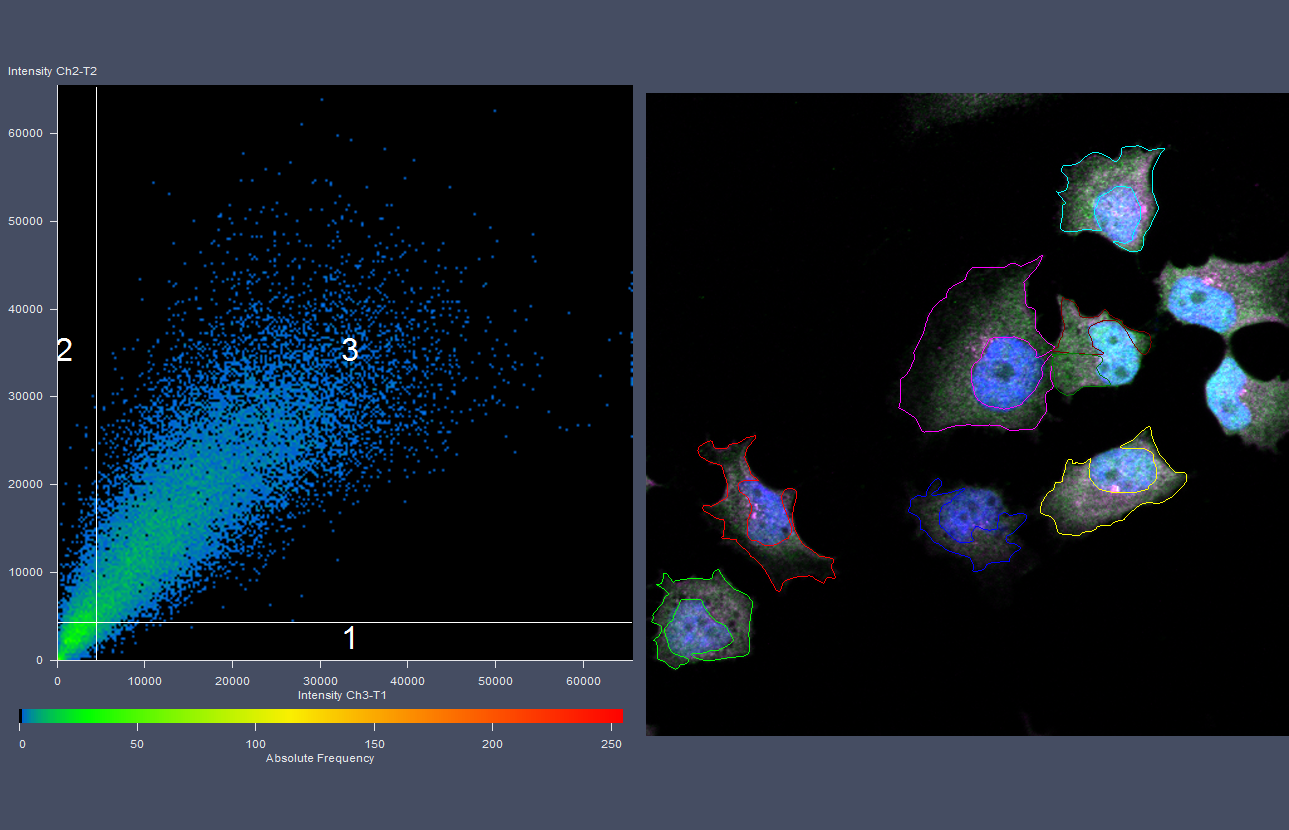
**

**#113
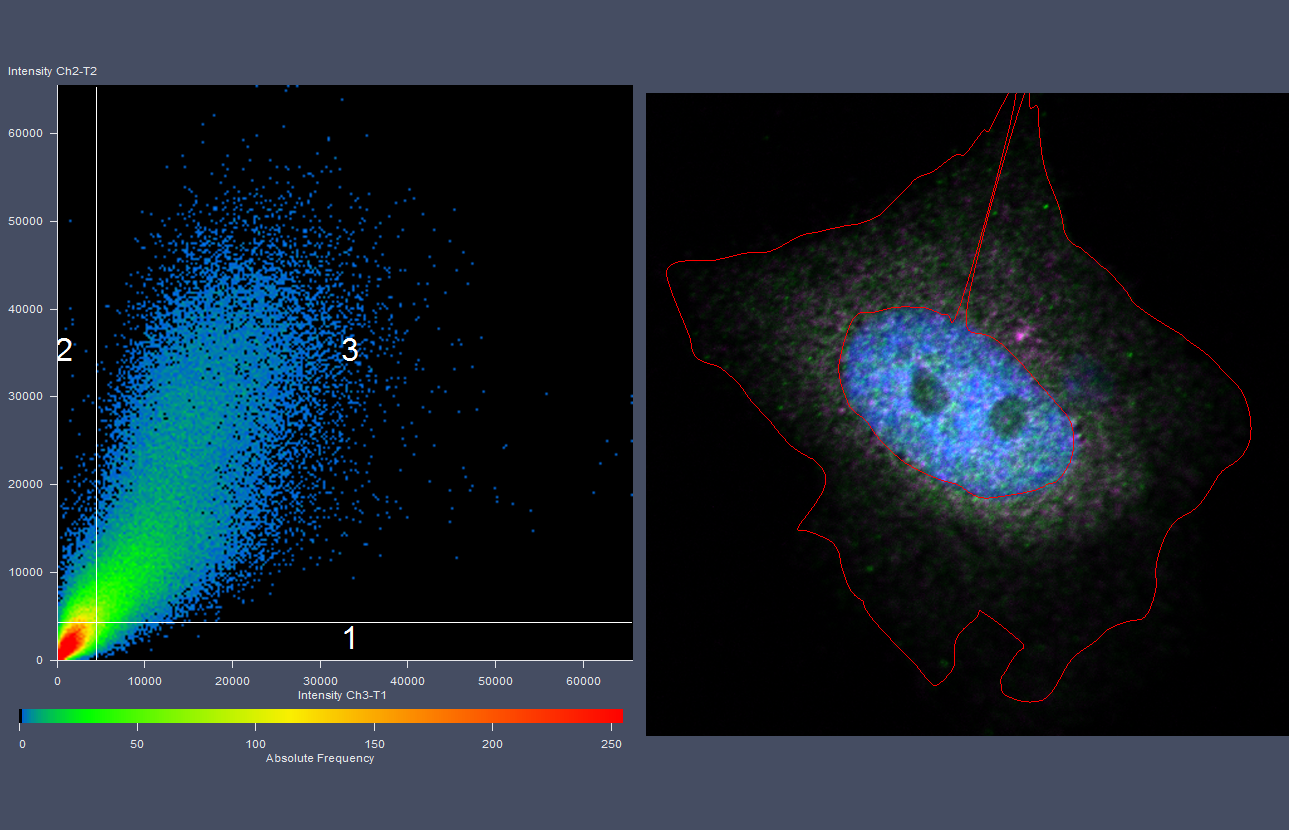
**

**#114
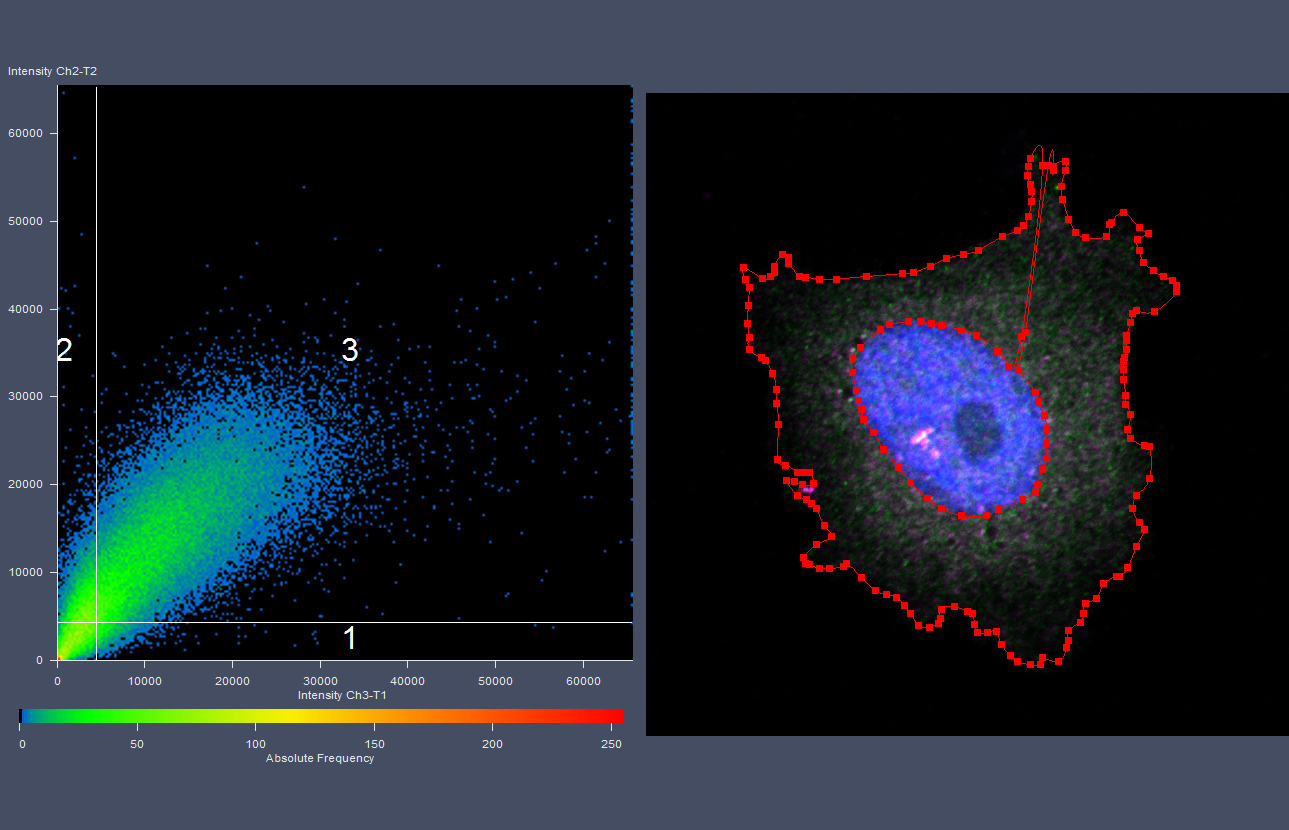
**

**#115
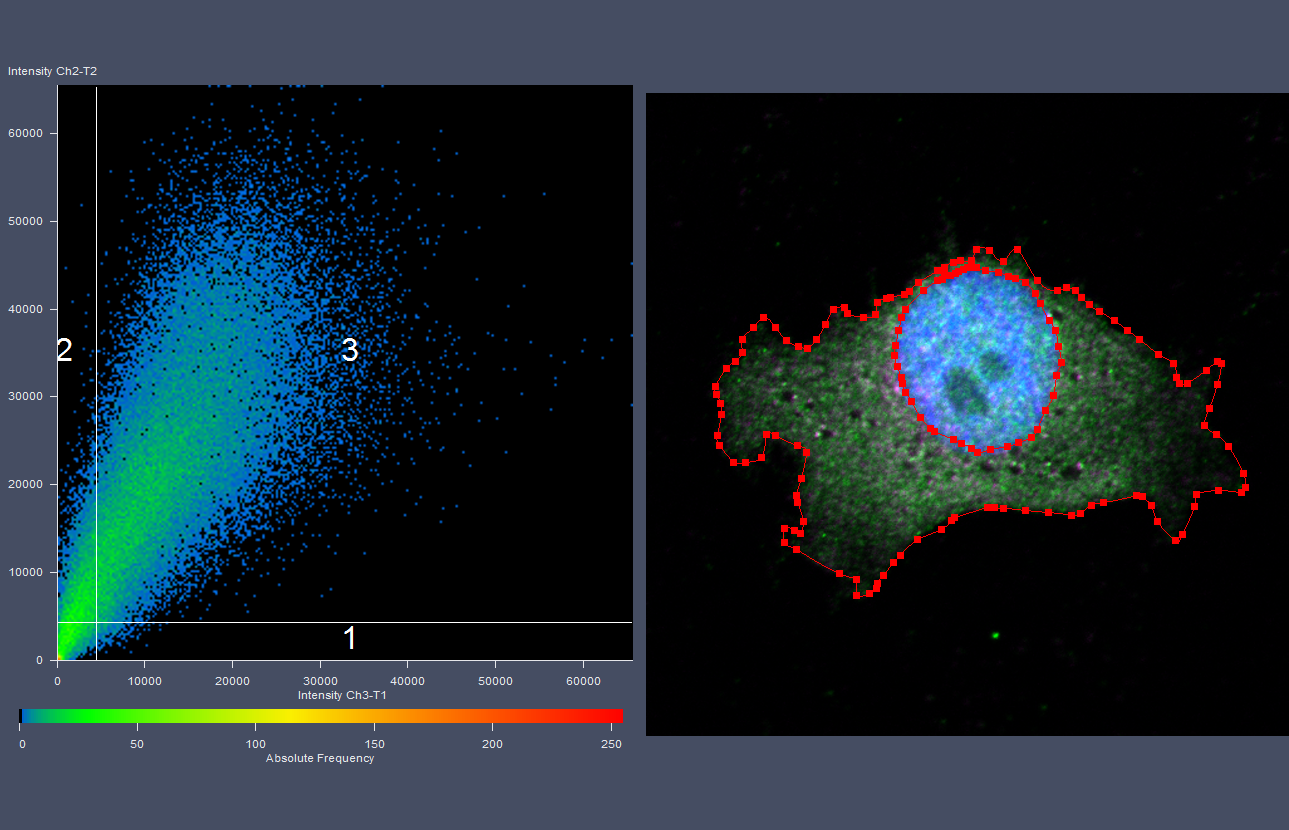
**

**#116-117**

**
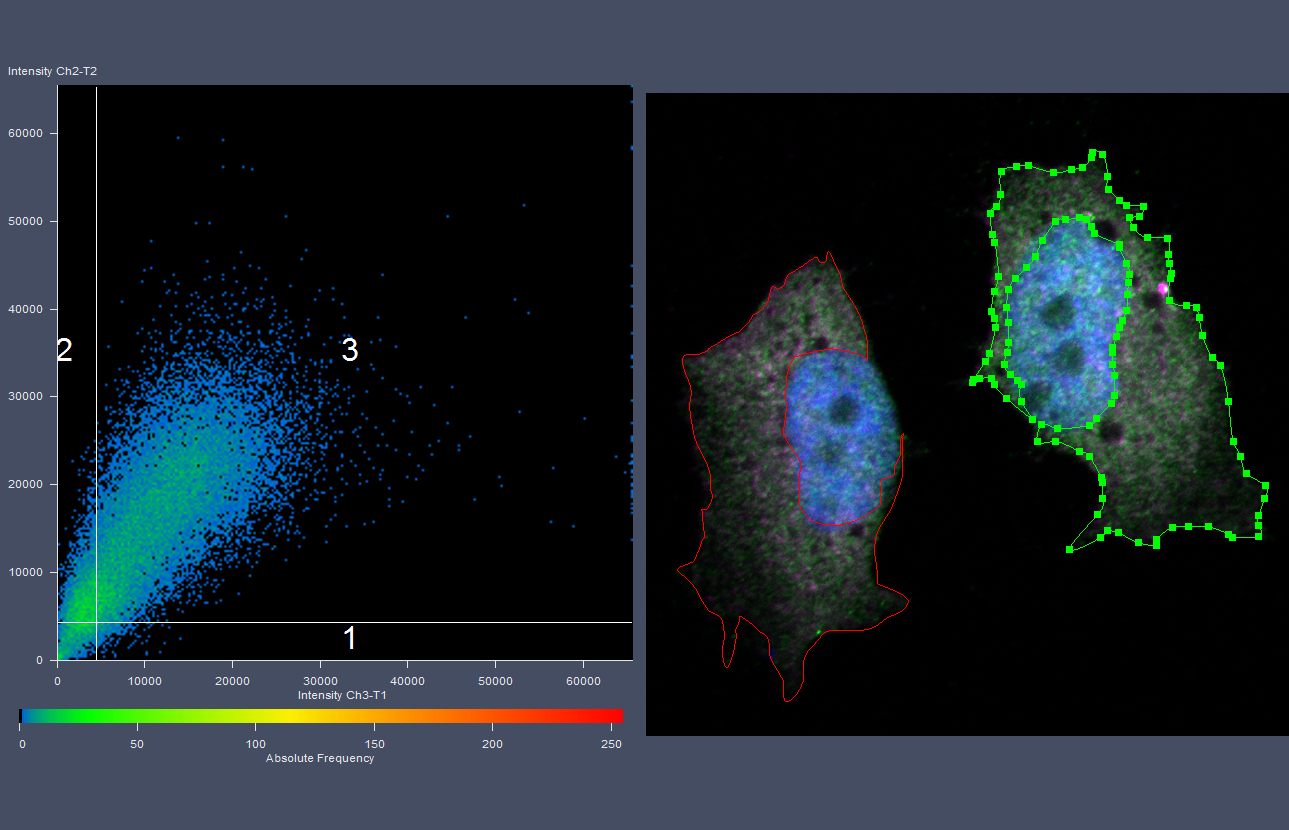
**

| **Overlap Coefficient** | **Correlation R** |  |  |  |  |
| --- | --- | --- | --- | --- | --- |
| 0,86 | 0,44 |  |  |  |  |
| 0,91 | 0,66 |  |  |  |  |
| 0,83 | 0,41 |  |  |  |  |
| 0,91 | 0,69 |  |  |  |  |
| 0,86 | 0,53 |  |  |  |  |
| 0,91 | 0,59 |  |  |  |  |
| 0,84 | 0,48 |  |  |  |  |
| 0,9 | 0,54 |  |  |  |  |
| 0,93 | 0,6 |  |  |  |  |
| 0,92 | 0,53 |  |  |  |  |
| 0,88 | 0,5 |  |  |  |  |
| 0,94 | 0,72 |  |  |  |  |
| 0,94 | 0,73 |  |  |  |  |
| 0,96 | 0,81 |  |  |  |  |
| 0,93 | 0,64 |  |  |  |  |
| 0,91 | 0,64 |  |  |  |  |
| 0,92 | 0,75 |  |  |  |  |
| 0,89 | 0,59 |  |  |  |  |
| 0,91 | 0,68 |  |  |  |  |
| 0,89 | 0,65 |  |  |  |  |
| 0,82 | 0,49 |  |  |  |  |
| 0,85 | 0,43 |  |  |  |  |
| 0,95 | 0,77 |  |  |  |  |
| 0,93 | 0,76 |  |  |  |  |
| 0,9 | 0,69 |  |  |  |  |
| 0,94 | 0,8 |  |  |  |  |
| 0,88 | 0,6 |  |  |  |  |
| 0,89 | 0,52 |  |  |  |  |
| 0,91 | 0,61 |  |  |  |  |
| 0,86 | 0,56 |  |  |  |  |
| 0,9 | 0,63 |  |  |  |  |
| 0,92 | 0,69 |  |  |  |  |
| 0,95 | 0,57 |  |  |  |  |
| 0,96 | 0,82 |  |  |  |  |
| 0,93 | 0,76 |  |  |  |  |
| 0,89 | 0,64 |  |  |  |  |
| 0,9 | 0,52 |  |  |  |  |
| 0,9 | 0,68 |  |  |  |  |
| 0,92 | 0,58 |  |  |  |  |
| 0,86 | 0,55 |  |  |  |  |
| 0,92 | 0,71 |  |  |  |  |
| 0,9 | 0,58 |  |  |  |  |
| 0,9 | 0,67 |  |  |  |  |
| 0,94 | 0,65 |  |  |  |  |
| 0,94 | 0,68 |  |  |  |  |
| 0,92 | 0,76 |  |  |  |  |
| 0,93 | 0,57 |  |  |  |  |
| 0,92 | 0,7 |  |  |  |  |
| 0,93 | 0,57 |  |  |  |  |
| 0,9 | 0,66 |  |  |  |  |
| 0,94 | 0,65 |  |  |  |  |
| 0,95 | 0,61 |  |  |  |  |
| 0,89 | 0,57 |  |  |  |  |
| 0,81 | 0,52 |  |  |  |  |
| 0,92 | 0,66 |  |  |  |  |
| 0,86 | 0,5 |  |  |  |  |
| 0,92 | 0,75 |  |  |  |  |
| 0,89 | 0,52 |  |  |  |  |
| 0,91 | 0,62 |  |  |  |  |
| 0,94 | 0,69 |  |  |  |  |
| 0,89 | 0,63 |  |  |  |  |
| 0,93 | 0,62 |  |  |  |  |
| 0,94 | 0,64 |  |  |  |  |
| 0,94 | 0,77 |  |  |  |  |
| 0,86 | 0,6 |  |  |  |  |
| 0,91 | 0,73 |  |  |  |  |
| 0,93 | 0,59 |  |  |  |  |
| 0,75 | 0,43 |  |  |  |  |
| 0,82 | 0,63 |  |  |  |  |
| 0,86 | 0,61 |  |  |  |  |
| 0,93 | 0,64 |  |  |  |  |
| 0,91 | 0,57 |  |  |  |  |
| 0,92 | 0,67 |  |  |  |  |
| 0,92 | 0,62 |  |  |  |  |
| 0,91 | 0,59 |  |  |  |  |
| 0,88 | 0,51 |  |  |  |  |
| 0,91 | 0,64 |  |  |  |  |
| 0,91 | 0,61 |  |  |  |  |
| 0,92 | 0,74 |  |  |  |  |
| 0,85 | 0,59 |  |  |  |  |
| 0,92 | 0,68 |  |  |  |  |
| 0,88 | 0,64 |  |  |  |  |
| 0,92 | 0,59 |  |  |  |  |
| 0,96 | 0,81 |  |  |  |  |
| 0,93 | 0,74 |  |  |  |  |
| 0,95 | 0,77 |  |  |  |  |
| 0,91 | 0,61 |  |  |  |  |
| 0,89 | 0,62 |  |  |  |  |
| 0,92 | 0,75 |  |  |  |  |
| 0,96 | 0,8 |  |  |  |  |
| 0,92 | 0,72 |  |  |  |  |
| 0,95 | 0,82 |  |  |  |  |
| 0,94 | 0,82 |  |  |  |  |
| 0,92 | 0,76 |  |  |  |  |
| 0,93 | 0,64 |  |  |  |  |
| 0,92 | 0,64 |  |  |  |  |
| 0,92 | 0,68 |  |  |  |  |
| 0,91 | 0,6 |  |  |  |  |
| 0,92 | 0,73 |  |  |  |  |
| 0,94 | 0,81 |  |  |  |  |
| 0,95 | 0,75 |  |  |  |  |
| 0,87 | 0,57 |  |  |  |  |
| 0,81 | 0,61 |  |  |  |  |
| 0,88 | 0,67 |  |  |  |  |
| 0,92 | 0,77 |  |  |  |  |
| 0,93 | 0,68 |  |  |  |  |
| 0,88 | 0,59 |  |  |  |  |
| 0,96 | 0,82 |  |  |  |  |
| 0,93 | 0,7 |  |  |  |  |
| 0,88 | 0,63 |  |  |  |  |
| 0,92 | 0,47 |  |  |  |  |
| 0,93 | 0,6 |  |  |  |  |
| 0,88 | 0,7 |  |  |  |  |
| 0,89 | 0,68 |  |  |  |  |
| 0,89 | 0,62 |  |  |  |  |
| 0,89 | 0,64 |  |  |  |  |
| 0,89 | 0,62 |  |  |  |  |

Supplement: Supplementary file 4 — Supplementary Material 4 [file 40364_2025_815_MOESM4_ESM.docx]
